# The use of bivariate copulas for bias correction of reanalysis air temperature data

**DOI:** 10.1371/journal.pone.0216059

**Published:** 2019-05-08

**Authors:** Fakhereh Alidoost, Alfred Stein, Zhongbo Su

**Affiliations:** 1 Department of Earth Observation Science, Faculty of the ITC, University of Twente, Enschede, the Netherlands; 2 Department of Water Resources, Faculty of the ITC, University of Twente, Enschede, the Netherlands; Tongji University, CHINA

## Abstract

Air temperature data retrieved from global atmospheric models may show a systematic bias with respect to measurements from weather stations. This is a common concern in local climate studies. The current study presents two methods based upon copulas and Conditional Probability (CP) to predict bias-corrected mean air temperature in a data-scarce environment: CP-I offers a single conditional probability as a predictor, CP-II is a pixel-wise version of CP-I and offers spatially varying predictors. The methods were applied on daily air temperature in the Qazvin Plain, Iran that were collected at 24 weather stations and 150 ECMWF ERA-interim grid cells from a single month (June) over 11 years. We compared the methods with the commonly applied conditional expectation and conditional median methods. Leave-*k*-out cross-validation and correlation scores show that the new methods reduced the bias with 44–68% for the full data set and with 34–74% on a homogeneous subarea. We conclude that the two methods are able to locally improve ECMWF air temperatures in a data-scarce area.

## Introduction

Assessment of the impact of climate change in agricultural areas is primarily based upon changes in weather data such as air temperature [1]. In a data-scarce environment, i.e., where weather stations are sparse, additional data are required. The European Centre for Medium-range Weather Forecasts (ECMWF) provides gridded ERA-interim reanalysis weather data that are being used increasingly [2]. They are prone to uncertainty because of the coarse resolution of models and variability of model parameters in space and time [3,4]. When compared with the measurements from weather stations, their bias is often considerable [5], in particular, if those measurements serve as benchmarks from which any measurement errors are ignored.

In this study we use copulas. A copula is a joint distribution function, describing the dependence structure between two or more variables [6]. The joint distribution function is estimated using any distribution family that can be different from the marginal distribution family of the involved variables [7]. Copula-based methods have been developed to correct bias in dependent variables [8,9]. Recently, copula-based methods are applied for deriving bias-corrected weather data [10–12]. Mao et al. [12] investigated bias correction methods of daily precipitation data and showed that a copula-based bias correction performs better than quantile mapping. After estimating the joint distribution, several methods are available to obtain bias corrected values. Examples are the conditional median (CM) [13], the conditional expectation (CE) [13,14], and the simulation method [7, 15].

So far, copula-based methods have been applied mainly to precipitation time series, where bias-corrected values are obtained using the simulation method [10–12]. Little attention, however, has been given to bias correction of air temperature data in a data-scarce area. Our main focus of bias correction is based upon the construction of the dependence structure between measurements and ECMWF reanalysis data using a joint distribution. The distribution is initially estimated using copulas and is then used to reduce bias of ECMWF air temperatures at grid cells that are often lacking a measurement from a weather station in a data-scarce area. To reduce bias in ECMWF air temperatures at those grid cells, an important aspect is the spatial variation of the data.

This study aims to introduce two copula-based predictors based upon Conditional Probabilities (CP) taking care of the spatial variation of daily air temperatures in a data-scarce area. The definition of the predictors and their application in a data-scarce environment is the main novelty of our study. We evaluate the performance of the predictors comparing to conventional methods like CE and CM in an agricultural area in Iran.

The structure of the paper is as follows. Copula-based bias correction methods are introduced in the second section. Our application is introduced in the third section and the results are shown in the fourth section. This is followed by the discussion and conclusion in the last sections.

## Copula-based bias correction methods

The structural, one sided difference between a measured value from a weather station *x*, and an ECMWF reanalysis value *y* is defined as the bias in ECMWF reanalysis values. We assume that the data are observed from two spatio-temporal random variables *X* and *Y*. In our study, the basis of the copula-based bias correction is a distribution function that allows for modeling the dependence structure between *X* and *Y*. The purpose of bias correction is to obtain ${\hat{x}}_{0}$ that denotes a predicted value at an unvisited location. An unvisited location is an ECMWF grid point without a measurement.

We focus on a bivariate distribution *F*(*x*,*y*); it can be extended to higher dimensions if more than two variables are available. The bivariate case is useful if ancillary information is unavailable. Regarding our main objective, we aim to introduce copula-based predictors to obtain ${\hat{x}}_{0}$. We first, illustrate both the commonly applied predictors and introduces the new predictors and next, present the estimation of marginals and copulas.

### Prediction

The conditional expectation (CE), the conditional median (CM) and the simulation method are commonly applied methods to obtain ${\hat{x}}_{0}$. CE and CM are both optimal predictors, minimizing the mean squared prediction error and the mean absolute prediction error, respectively [16–17]. They obtain the bias-corrected value ${\hat{x}}_{0}$ as:

$$\mathrm{CE}:\;{\hat{x}}_{0}=E\left[X|Y=y_{0}\right]=\int {}_{x}x\cdot f\left(x|y_{0}\right)dx,$$ (1)

$$\mathrm{CM}:\;{\hat{x}}_{0}=F^{-1}(p|y_{0}),\;p=0.5,$$ (2)

where *f*(.|.) is conditional density distribution function, *F*^−1^ denotes the inverse transformation of the conditional distribution *F*(.|.), and *p* is the conditional probability that determines the median. Both CE and CM are either an increasing or a decreasing function of the conditioning variable *Y* depending upon the sign of the dependence between *X* and *Y* (cf. S1 appendix). Therefore, the variation of bias-corrected values follows the variation of ECMWF reanalysis values rather than those of the measurements; this will be further illustrated in Results section.

The third method is the simulation method. It obtains *m* bias-corrected values by generating *m* conditional probabilities *p* on [0, 1] as:

$${\hat{x}}_{0,k}=F^{-1}(p_{k}|y_{0}),k=1,\ldots ,m.$$ (3)

Note that the mean of $\left\{{\hat{x}}_{0,1},\ldots ,{\hat{x}}_{0,m}\right\}$ provides a single value ${\hat{x}}_{0}$ and that both the value of *m* and the simulations themselves influence the results. For a large *m*, the results of this method are equal to the results of CE [12]. In case of choosing the median of $\left\{{\hat{x}}_{0,1},\ldots ,{\hat{x}}_{0,m}\right\}$, this also applies to CM.

For CE, the mean value of the distribution *F*(*x*|*y*_0_) is selected as ${\hat{x}}_{0}$, whereas for CM, this is the median value of the distribution. We may question whether mean and median values best suit bias-corrected air temperatures. In the following, two new methods are introduced to obtain a conditional probability which serves as a predictor.

CP-I and CP-II are the predictors, minimizing mean absolute bias (MAB) as:

$$MAB=\frac{1}{n}\sum_{i=1}^{i=n}\left|x_{i}-F^{-1}(p|y_{i})\right|,$$ (4)

where for CP-I, *n* = *N* and equals the total number of observations, whereas for CP-II, *n* = *M* ≪ *N* and equals the number of observations at the nearest *M* locations to *x*_0_. The conditional probability *p* is iteratively estimated based upon minimizing MAB in (4) resulting in the optimal p* value. The bias-corrected value ${\hat{x}}_{0}$ then equals:

$${\hat{x}}_{0}=F^{-1}(p|y_{0}),p=p^{*}.$$ (5)

For CP-I, the conditional probability p* is constant for all unvisited locations, e.g. *F*(*x*_0_|*y*_0_) = p*. Therefore, similar to CE and CM, CP-I is either an increasing or a decreasing function of the conditioning variable, depending upon the sign of the dependence (cf. S1 appendix). For CP-II, the optimal conditional probability depends upon unvisited location and is denoted now by $p_{0}^{*}$, e.g. $F(x_{0}|y_{0})=p_{0}^{*}$.

Next we formulate the equations using copulas and investigate the use of copulas for the construction of distribution functions. A good description of copulas is available from [7]. According to Sklar’s theorem, *F*(*x*,*y*) is equal to a copula *C*(*u*,*v*) of two uniformly distributed variables *u* = *F*_*X*_(*x*) and *v* = *F*_*Y*_(*y*), where *F*_*X*_ and *F*_*Y*_ are marginal distributions. It can be shown that *F*(*x*|*y*) = *C*(*u*|*v*) and the predictors are rewritten as:

$$\mathrm{CE}:\;{\hat{x}}_{0}=\int_{0}^{1}\;F_{X}^{-1}(u)\times c(u|V=v_{0})du,$$

$$\mathrm{CM}:\;{\hat{x}}_{0}=F_{X}^{-1}(C^{-1}(p|V=v_{0})),p=0.5,$$

$$\mathrm{CP}:\;MAB=\frac{1}{n}\sum_{i=1}^{i=n}\left|x_{i}-F_{X}^{-1}(C^{-1}(p|V=v_{i}))\right|,\;{\hat{x}}_{0}=F_{X}^{-1}(C^{-1}(p|V=v_{0})),p=p^{*}.$$

where $F_{X}^{-1}$ denotes the inverse transformation of the marginal cumulative distribution function *F*_*X*_, *v* is marginal probability i.e. *v* = *F*_*Y*_(*y*), *c*(.|.) is the conditional density copula, and *C*(.|.) is the conditional cumulative copula (cf. appendix 2).

Before introducing estimation of the distribution functions, we now explain the implementation of CP-I and CP-II to identify the optimal conditional probability. Initially, a probability *p* = 0.01 is chosen and MAB is obtained from Eq (4). Then the probability *p* increases with steps of 0.01 until *p* = 1. We select the probability p* that results into the lowest MAB. Finally, the bias-corrected value ${\hat{x}}_{s_{0}}$ is obtained from Eq (5). The choice for the initial probability and for a step value equal to 0.01 are based upon our experience on the variable of interest and uncertainty sources. We compare this value using a sensitivity analysis on the mean absolute prediction error to assess the effect of choosing larger or smaller increment values i.e. 0.1 or 0.001; the assessments are reported in the Results section below. Note that CP-I is implemented only once, whereas CP-II is implemented at each unvisited location separately and therefore has a higher computational cost.

### Estimation

In practice, finite samples on *X* and *Y* are observed in space and time without replication. Therefore, the joint distribution *F*(*x*,*y*) is estimated using the assumption of stationarity (in space or time), i.e. marginal distributions and dependence structure between *X* and *Y* are irrespective of location or time. In the literature, reviewed in introduction, the current bias correction methods have been applied to climate time series assuming temporal stationarity. Hence, removing autocorrelation and heteroscedasticity that may exist in any climate time series, is necessary for any estimation procedure [10]. To achieve our main objective, we apply a bias correction to predict ${\hat{x}}_{0}$ at an unvisited location in space, separately at each day of time series.

Estimation of theoretical marginal distributions may affect the estimation of the copula parameter and consequently the selection of the copula family. Therefore, we use empirical marginal distributions. By means of kernel density estimation, a continuous approximation of the marginal distribution are obtained under the assumption of stationary [18]. We evaluate this assumption using a regression analysis and the auto-correlation function (See S3 Appendix). The choice of the method to estimate empirical marginal probability is not unique and a more specific sensitivity analysis might help to show the effects of other marginal distribution functions on the results. This, however, is outside the scope of the study.

The bivariate copula *C* can be determined using several copula families. We assume spatial stationarity and evaluate the assumption using a co-correlation function (See S3 Appendix). We consider the Gaussian, Student’s *t*, Clayton, Gumbel and Frank families [19–22]. Other copula families like the Farlie-Gumbel-Morgenstern and Joe families [7] were not considered as obtaining the inverse of the conditional copula distribution and the implementation of partial derivatives may lead to computational problems [13]. The *p* value of the null hypothesis of bivariate independence is obtained based upon the statistical test for independence developed by [23]. The parameter of the bivariate copula is related to correlation between variables (Table 1).

**Table 1:** Five families of copulas estimated on each day in this study. The best fitting family is selected according to the lowest value of Akaike Information Criteria (AIC).

Index	Name	*C*_*θ*_*(u*,*v)*	Property index
**1**	**Gaussian**	$\emptyset_{R}(\emptyset^{-1}(u),\emptyset^{-1}(v));R=\left[\begin{array}{cc} 1 & \theta \\ \theta & 1 \end{array}\right]$	1, 2, 6
**2**	**Student’s *t***	$t_{R,\vartheta }(t_{\vartheta }^{-1}(u),t_{\vartheta }^{-1}(v));\;R=\left[\begin{array}{cc} 1 & \theta \\ \theta & 1 \end{array}\right];\vartheta =degree\;of\;freedom$	1, 2, 6
**3**	**Clayton**	${\left[max\{(u^{\theta }+v^{\theta }-1),0\}\right]}^{\frac{-1}{\theta }}$	1, 2,4,5,6
**4**	**Gumbel**	$exp(-{\left[{\left(-lnu\right)}^{\theta }+{\left(-lnv\right)}^{\theta }\right]}^{\frac{1}{\theta }})$	1,2,3,6
**5**	**Frank**	$\frac{-1}{\theta }ln(1+\frac{\left(e^{-\theta u}-1)(e^{-\theta v}-1\right)}{e^{-\theta }-1})$	1,2,6
**1**	**Property**	Permutation symmetry	
**2**		Symmetry about medians	
3		Extreme value copula	
**4**		Lower tail dependence	
**5**		Upper tail dependence	
**6**		Extendibility to multivariate copula	

We estimate the parameter for each family using maximum likelihood and a starting value obtained by Kendall’s τ correlation [7, 24]. Then the best family for C is the one that minimizes Akaike’s Information Criteria (AIC) [25]. The p values of the null hypothesis that the dependence structure is well represented by this family are obtained using 100 bootstrap runs based upon the Cramér–von Mises statistic $S_{n}^{\left(B\right)}$ for the Gaussian, Clayton, Gumbel and Frank families [26], and based upon the White statistic for the Student’s t family [27]. This number of bootstrap runs is relatively small, but a larger number would substantially increase the computational cost.

### Evaluation

We evaluate the performance of the proposed predictors using errors and correlations between measured and bias-corrected values. To obtain errors, we apply the leave-*k*-out cross-validation [28]. The bias-corrected value ${\hat{x}}_{s,t}$ at day *t* and location *s* is obtained by leaving *k* observations out for the same day of the year in *k* successive years and using the reminder of the observations. The mean absolute error *MAE*_*s*,*t*_ is defined as:

$${MAE}_{s,t}=\frac{1}{k}\sum_{i=1}^{k}\left|x_{s,t,i}-{\hat{x}}_{s,t,i}\right|.$$ (6)

We define three criteria based upon the mean absolute errors to compare the presented methods at *N* weather stations and *T* days:

$$MAE=\frac{1}{T}\sum_{t=1}^{T}(\frac{1}{N}\sum_{s=1}^{N}{MAE}_{s,t}).$$ (7)

$$SES=\sum_{s=1}^{N}\left(rank\left(\frac{1}{T}\sum_{t=1}^{T}MAE_{s,t}\right)\right),$$ (8)

$$TES=\sum_{t=1}^{T}\left(rank\left(\frac{1}{N}\sum_{s=1}^{N}MAE_{s,t}\right)\right).$$ (9)

where the *MAE* is the overall mean absolute error, *SES* and *TES* are spatial and temporal error scores [4], $\frac{1}{T}\sum_{t=1}^{T}{MAE}_{s,t}$ and $\frac{1}{N}\sum_{s=1}^{N}{MAE}_{s,t}$ are spatial and temporal mean absolute errors, respectively. A low value of a criterion indicates a good performance.

To obtain correlations, the bias-corrected value ${\hat{x}}_{s,t}$ at day *t* and location *s* is obtained using all observations. The temporal correlations *r*_*s*_ at location *s* and the spatial correlations *r*_*t*_ at day *t* are used to evaluate the temporal and spatial variations of the bias corrected values:

$$r_{s}=corr\left(\left\{{\hat{x}}_{s,t},\ldots ,{\hat{x}}_{s,T}\right\},\left\{x_{s,t},\ldots ,x_{s,T}\right\}\right),s=1,\ldots ,N,$$ (10)

$$r_{t}=corr(\left\{{\hat{x}}_{1,t},\ldots ,{\hat{x}}_{N,t}\},\{x_{1,t},\ldots ,x_{N,t}\right\}),t=1,\ldots ,T.$$ (11)

We define two criteria to compare the methods based upon the correlations as:

$$SCS=\sum_{s=1}^{N}\left(rank(r_{s})\right),$$ (12)

$$TCS=\sum_{t=1}^{T}\left(rank(r_{t})\right),$$ (13)

where *rank*(.) returns the rank of a number within a set of numbers, *SCS* and *TCS* are spatial and temporal correlation scores, respectively. A high value of *SCS* and *TCS* indicates a good performance.

The study was carried out in R using the packages VineCopula [24], gstat [29], and copula [30].

## Application

**Fig 1:**
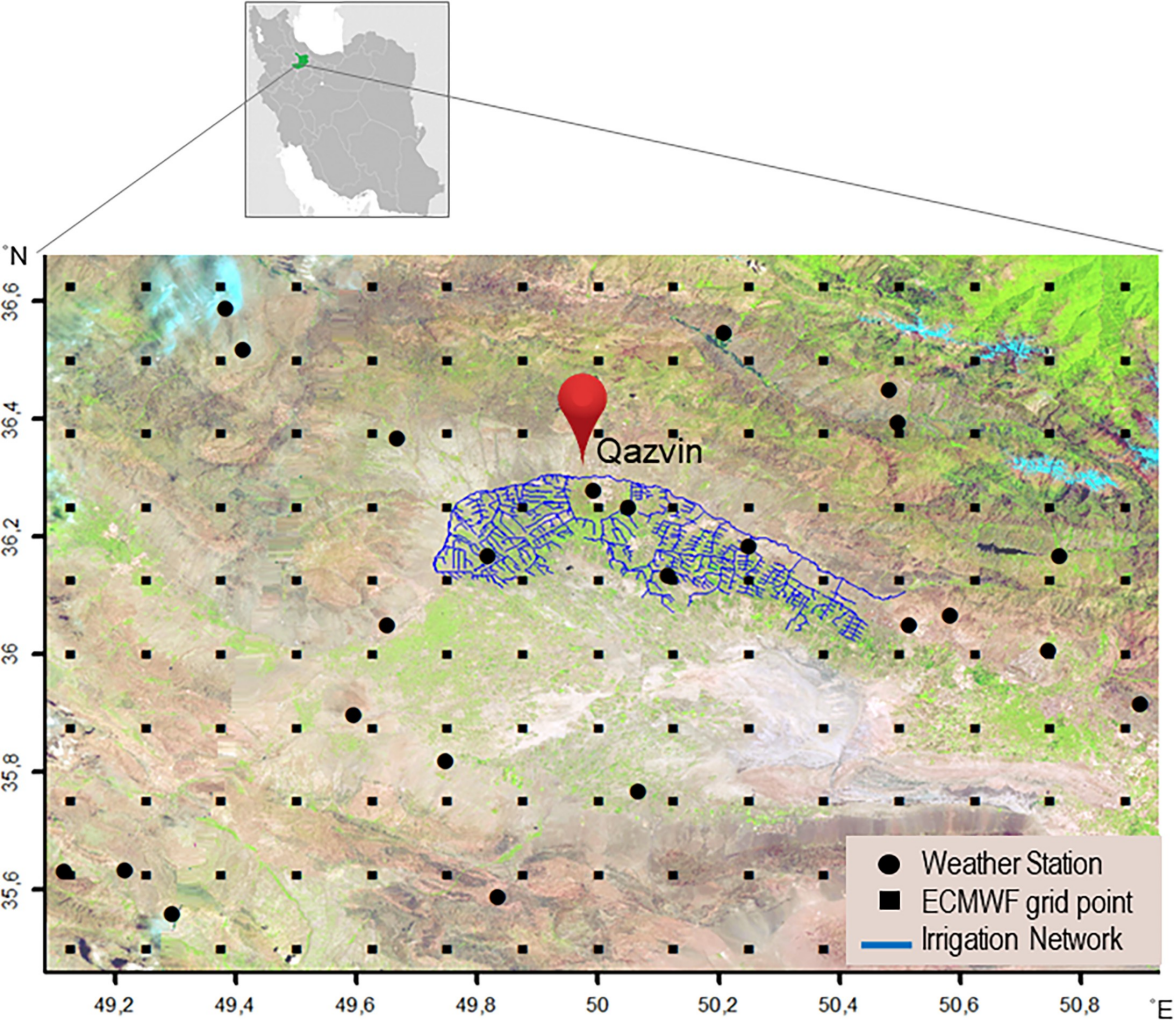
The irrigation network in Qazvin Plain, Iran. The area includes 24 weather stations and a sample subset of 10 × 15 grid cells of ECMWF dataset. The background image is obtained by Landsat 8 RGB bands.

The bias correction methods are compared in an actual study on air temperature data in the Qazvin irrigation network, Iran (Fig 1). The study area extends from 35.44° to 36.68° latitudes (N) and from 49.09° to 50.92° longitudes (E) and it includes 24 weather stations (Fig 1). The Qazvin area is one of the oldest agricultural areas in the world where maize, wheat, barley and orchards are the dominating crops. Besides it contains urban areas and natural vegetation. The European Centre for Medium-Range Weather Forecasts (ECMWF) provides reanalysis data at a wide range of spatial resolutions, e.g. regular and rotated lat/lon grids, and reduced Gaussian grid. For the dissemination, air temperature is bi-linearly interpolated to a 0.125° lat/lon grid at three hourly intervals. A grid of 10 × 15 cells covers the study area (Fig 1). ERA-Interim provides widely used global atmospheric reanalysis data [3]. The reanalysis air temperatures are retrieved for 150 grid cells at a 0.125° lat/lon resolution from the ERA-Interim data assimilation system.

**Fig 2:**
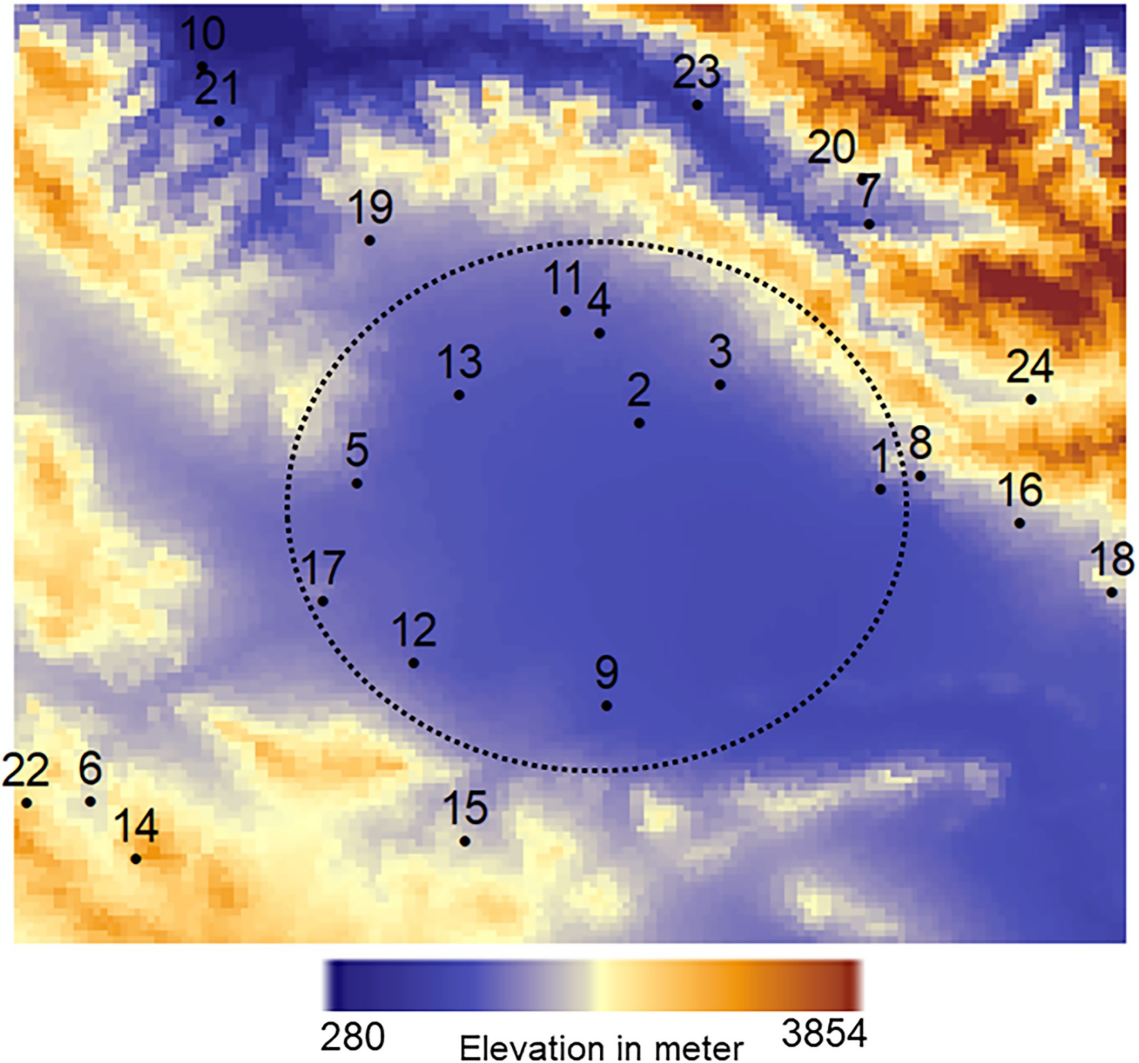
Elevations (m) are covariates for air temperature in the CP-II including covariate. It is obtained by MODIS product at a 1km spatial resolution. Location and index of the weather stations are shown in this figure. We applied the presented methods on a complete set of 24 weather stations as well as a subset of ten stations where the spatial variation of elevation is more homogenous i.e. the area indicated by circle.

The NASA Land Processes Distributed Active Archive Centre (LPDAAC) provides the Moderate Resolution Imaging Spectroradiometer (MODIS) products. The MOD03 product provides per-pixel digital elevation model values in a sequence of swath-based products at 5-minute increments. This resulted in elevations at a 1km spatial resolution (Fig 2). The dependence structure between air temperature and elevation does not follow the lapse-rate law (cf. S4 Fig). To extend the bivariate joint distribution to higher dimensions by including elevation, we investigate whether considering elevation improves the results of the bias correction methods (cf. Evaluation and comparison section). In our study, the dependence structures between the reanalysis values and measured values are studied in a relatively small and homogenous area and are thus likely to change spatially in a stationary way. An exception concerns the mountains in North-Eastern part of the study area (Fig 2). To evaluate the potential effect of spatial non-stationarity, we applied the presented methods both on a complete set of 24 weather stations and on a subset of ten stations where the spatial variation of elevation is more homogenous (Fig 2).

**Fig 3:**
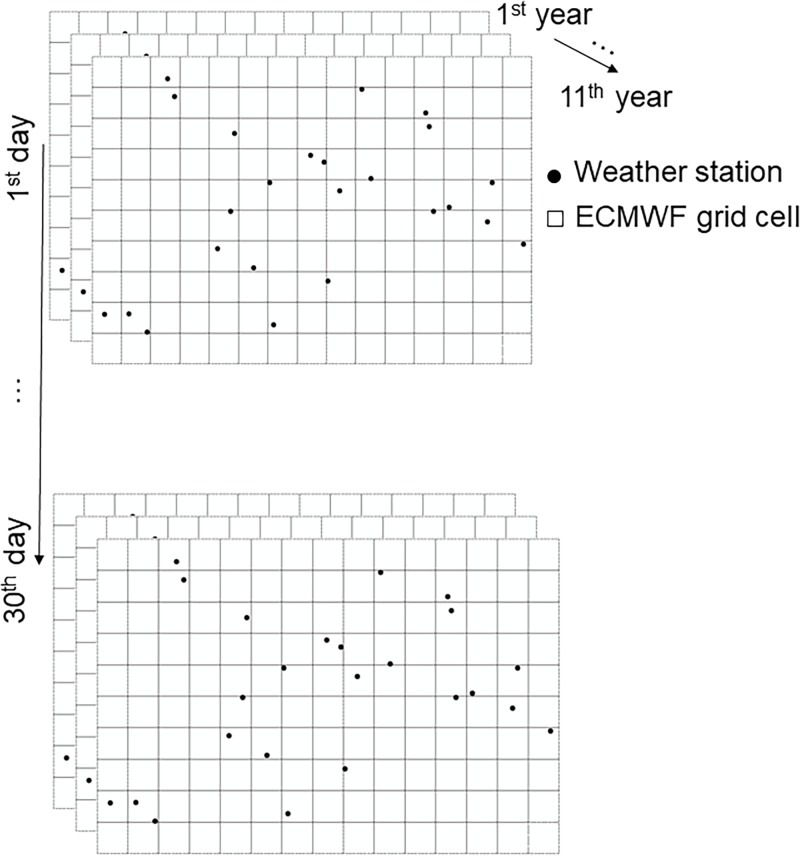
The data frame. Daily air temperatures in June are available at 24 stations and 150 grid cells of ECMWF during 11 years. We apply the presented methods separately on each day. A copula is the daily bivariate distribution function between measurements from a weather station and the reanalysis data from ECMWF.

Daily mean air temperatures in June from 2004 to 2014 are selected (Fig 3) as June is an important month in the crop calendar [31]. The copula we consider in our paper is the daily bivariate distribution function of the measurements from a weather station and the reanalysis data from ECMWF. We pool air temperatures for the same days across 11 years. This results into 150 grid cells×11 years = 1650 reanalysis values on each day in June. In addition, there are 24 stations×11 years = 264 measured values. This number of measured values can differ between the different weather stations due to a varying number of missing values (cf. Table 2). The bias-corrected daily air temperature is obtained at unvisited locations in June 2014, applied on each day, separately. In this way, the methods are tested 30 times. We realize that in doing so, effects of non-stationarity may exist due to climate change. For this time series of 11 years, however, we felt safe to ignore those effects.

**Table 2:** The *p* values and selected family on each day in June. Number of data denotes number of available data for fitting purposes and equals to the number of measurements from weather stations during years 2004 to 2014 on each day in June. The *p* value-1 is obtained under the null hypothesis of bivariate independence. The copula families are: N = Gaussian, T = Student’s *t*, C = Clayton, G = Gumbel and F = Frank. The *p* values-2 are obtained by the Cramér–von Mises statistic $S_{n}^{\left(B\right)}$.

Day	Number of data	*p* value-1	Selected family	*p* value-2
1	226	0.00	G	0.42
2	224	0.00	N	0.62
3	226	0.00	G	0.48
4	226	0.00	G	0.58
5	226	0.00	T	1.00
6	226	0.00	G	0.40
7	226	0.00	N	0.44
8	225	0.00	T	1.00
9	226	0.00	G	0.34
10	226	0.00	G	0.26
11	226	0.00	G	0.36
12	226	0.00	N	0.62
13	226	0.00	N	0.44
14	226	0.00	N	0.64
15	226	0.00	G	0.44
16	226	0.00	G	0.52
17	226	0.00	G	0.46
18	226	0.00	G	0.44
19	226	0.00	F	0.25
20	226	0.00	G	0.34
21	226	0.00	G	0.30
22	226	0.00	G	0.79
23	225	0.00	G	0.36
24	226	0.00	G	0.54
25	226	0.00	G	0.75
26	226	0.00	G	0.68
27	226	0.00	N	0.50
28	226	0.00	F	0.44
29	226	0.00	F	0.60
30	225	0.00	G	0.54

The weather stations are categorized into three types based upon the instrument and temporal frequency of the measurements (cf. S1 Table). Air temperature is measured by a thermometer in the synoptic and climatology type1 stations and it is measured by a thermograph in the climatology type2 stations. The time series of the air temperature at the climatology type2 stations e.g. stations 11, 13 and 21 (cf. S1–S3 Figs) reveals that the quality of the measurements is low. The synoptic stations are supposed to provide more precise measurements. In the next section, we report to which degree the results of the presented methods are affected by different qualities of the measurements at the three types of the stations.

**Fig 4:**
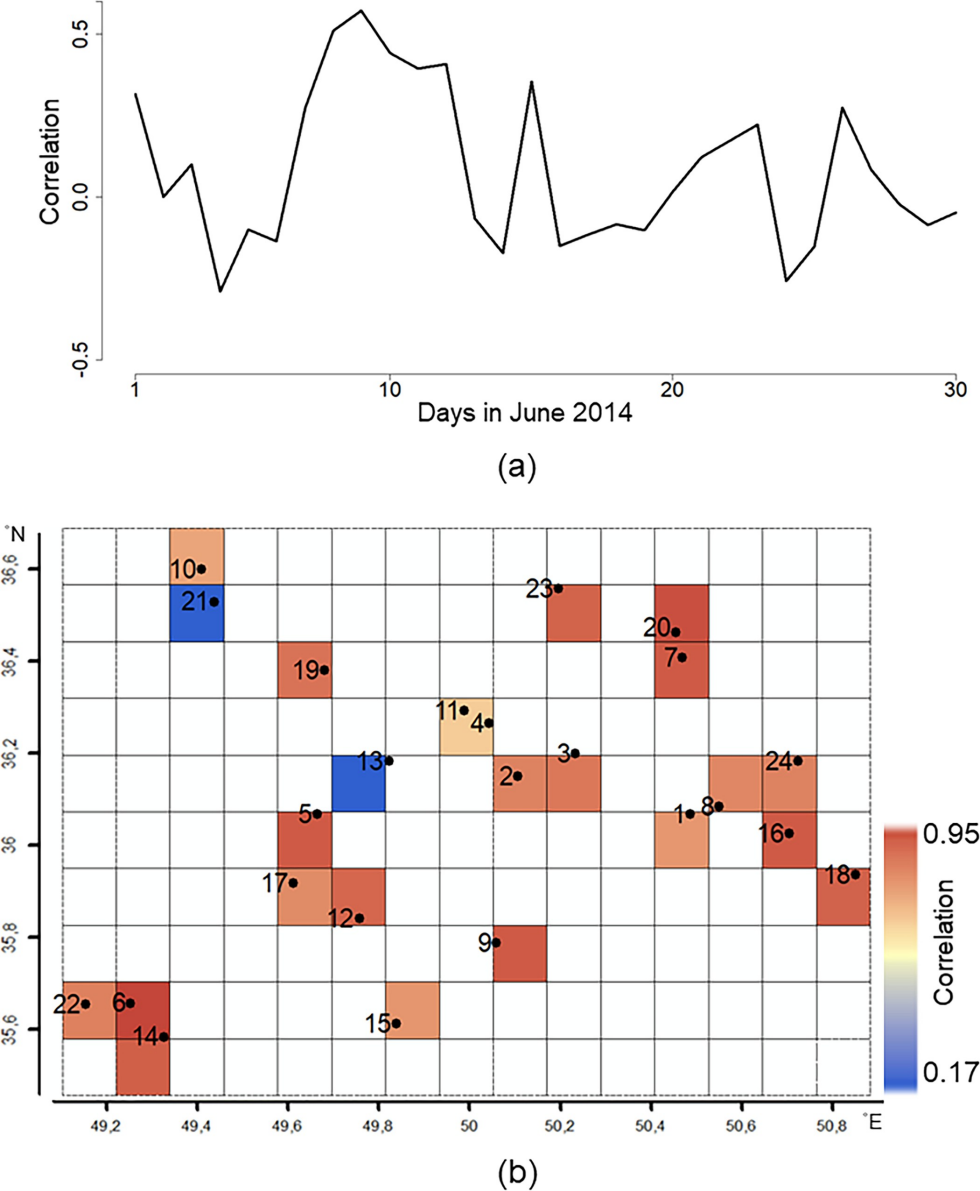
The correlation coefficient *r* between reanalysis data and measurements from weather stations a) on each day in June 2014, b) at each weather station. The numbers on the second figure denote the weather stations’ number.

To compare reanalysis values with measured values, each station is assigned to its nearest grid cell. Overestimation and underestimation of reanalysis data has been observed in June 2014 (S1–S3 Figs). Correlations *r*_*t*_ between reanalysis values and measured values in space are low at most days in June 2014 (Fig 4A). In addition, correlations *r*_*s*_ at the weather stations 13 and 21 are rather weak (Fig 4B).

## Results

### Marginal distributions and copulas

**Fig 5:**
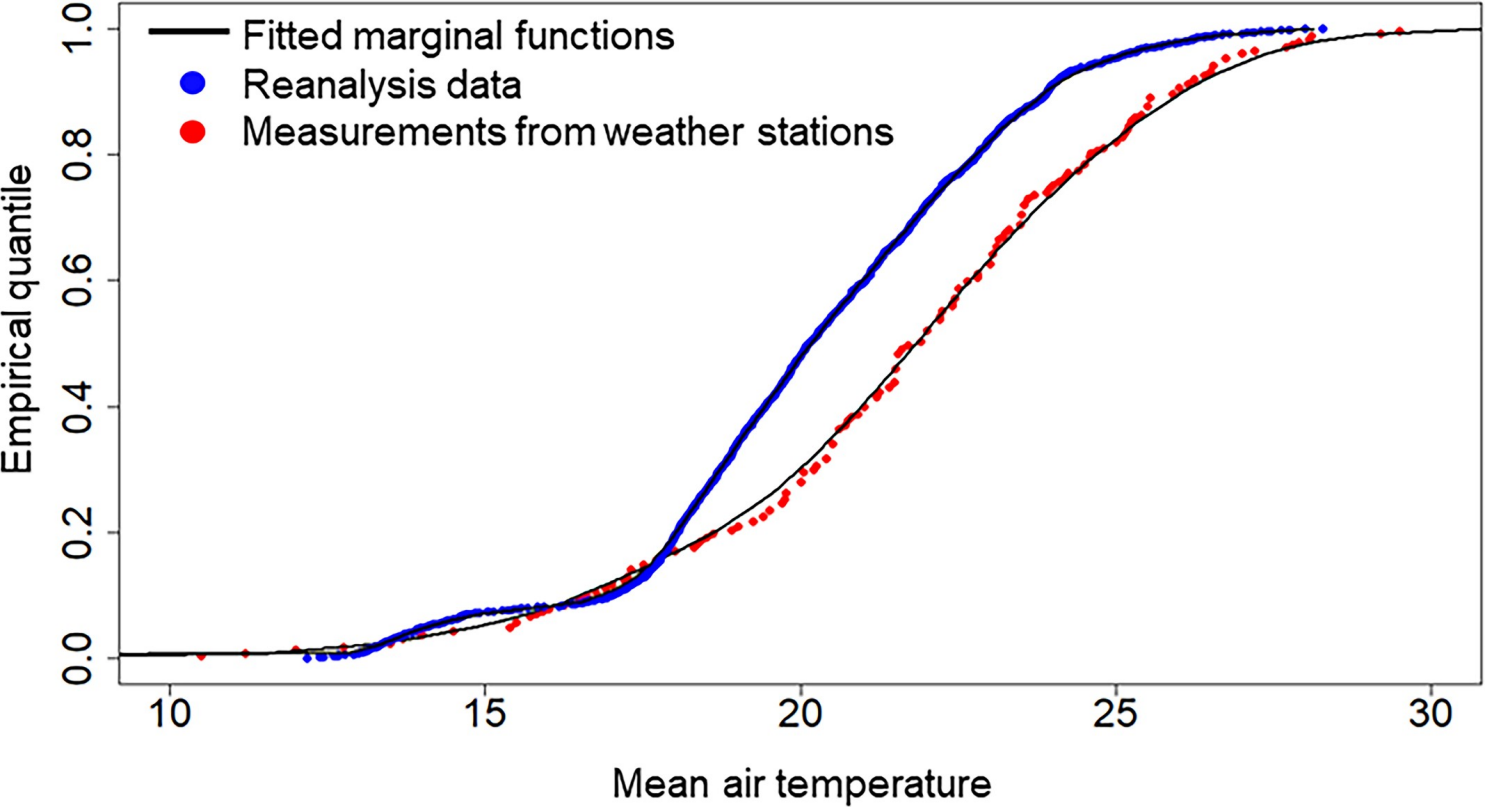
Empirical marginal probabilities at June 1^st^. Marginal probabilities are obtained using kernel density estimation on each day of June using eleven years series from 2004 to 2014 at 24 weather stations.

Fig 5 shows the fits of marginal distribution functions assuming spatial stationarity. S3 appendix presents the evaluation of this assumption on each day in June 2014.

The parameters of five copula families are estimated on each day of June assuming spatial stationarity. Appendix 3 further contains the evaluation of this assumption for copulas. Table 2 shows the number of data used for fitting. The *p* value of the null hypothesis of bivariate independence is zero, thus rejecting the null hypothesis (Table 2, third column). The best fitting family based upon the lowest AIC value turned out to be Gumbel family for 17 days in June. The *p* values of the Cramér–von Mises statistic $S_{n}^{\left(B\right)}$ were larger than 0.2 for all days (Table 2, last column), hence not rejecting the null hypothesis. We could safely assume that the best fitting family well describes the dependence structure.

### Evaluation and comparison

The optimal conditional probability obtained using CP-I, and the minimum and maximum of the optimal conditional probabilities obtained using CP-II on each day are given in Table 3. The conditional probability using CP-I clearly changes in time in the range of [0.30, 0.95]. For CP-II, the optimal conditional probability changes in time and space in the range of [0.02, 0.99], using *M =* 4. Influence of the choice of the increment value in CP-I is assessed using sensitivity analysis (cf. S5 Fig). It revealed that the uncertainty is higher using an increment value of 0.1, whereas for 0.001 no improvements were achieved.

**Table 3:** Optimal conditional probabilities. A single optimal conditional probability is obtained using CP-I for all unvisited locations on each day whereas using CP-II, it is obtained at each unvisited location and each day. The minimum and maximum of the optimal conditional probabilities obtained by CP-II are mentioned here.

Day	Optimal conditional probability in CP-I	Minimum and maximum optimal conditional probabilities in CP-II	
1	0.79	0.13	0.90
2	0.60	0.08	0.97
3	0.30	0.04	0.92
4	0.36	0.08	0.93
5	0.50	0.02	0.90
6	0.61	0.08	0.93
7	0.71	0.12	0.96
8	0.66	0.21	0.92
9	0.64	0.25	0.90
10	0.82	0.23	0.99
11	0.87	0.28	0.98
12	0.68	0.09	0.95
13	0.58	0.06	0.84
14	0.57	0.05	0.88
15	0.65	0.10	0.86
16	0.65	0.09	0.94
17	0.76	0.07	0.84
18	0.55	0.10	0.74
19	0.73	0.07	0.88
20	0.69	0.19	0.91
21	0.50	0.13	0.95
22	0.83	0.19	0.98
23	0.91	0.23	0.99
24	0.64	0.14	0.96
25	0.65	0.09	0.94
26	0.79	0.17	0.92
27	0.74	0.13	0.98
28	0.83	0.10	0.95
29	0.92	0.21	0.98
30	0.79	0.16	0.99

**Fig 6:**
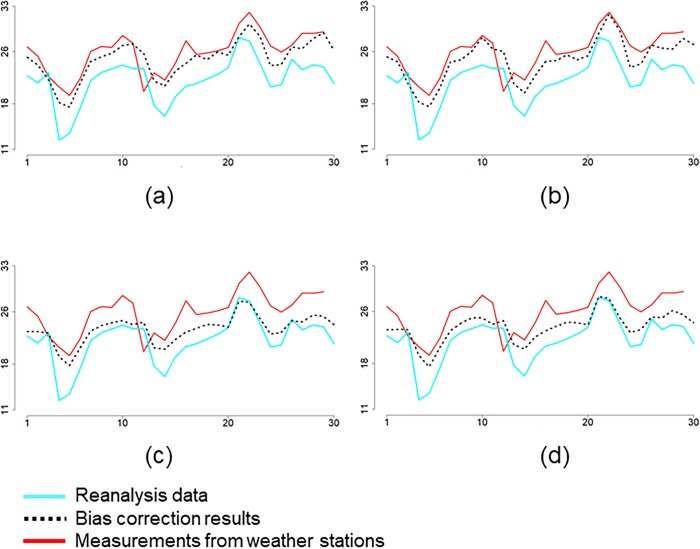
Time series of the mean air temperatures at first station in June 2014 obtained by the measurements, the reanalysis data, and the results of a) CP-I, b) CP-II, c) CE and d) CM. The vertical axis is daily mean air temperature in °C. The horizontal axis is days in June 2014.

**Fig 7:**
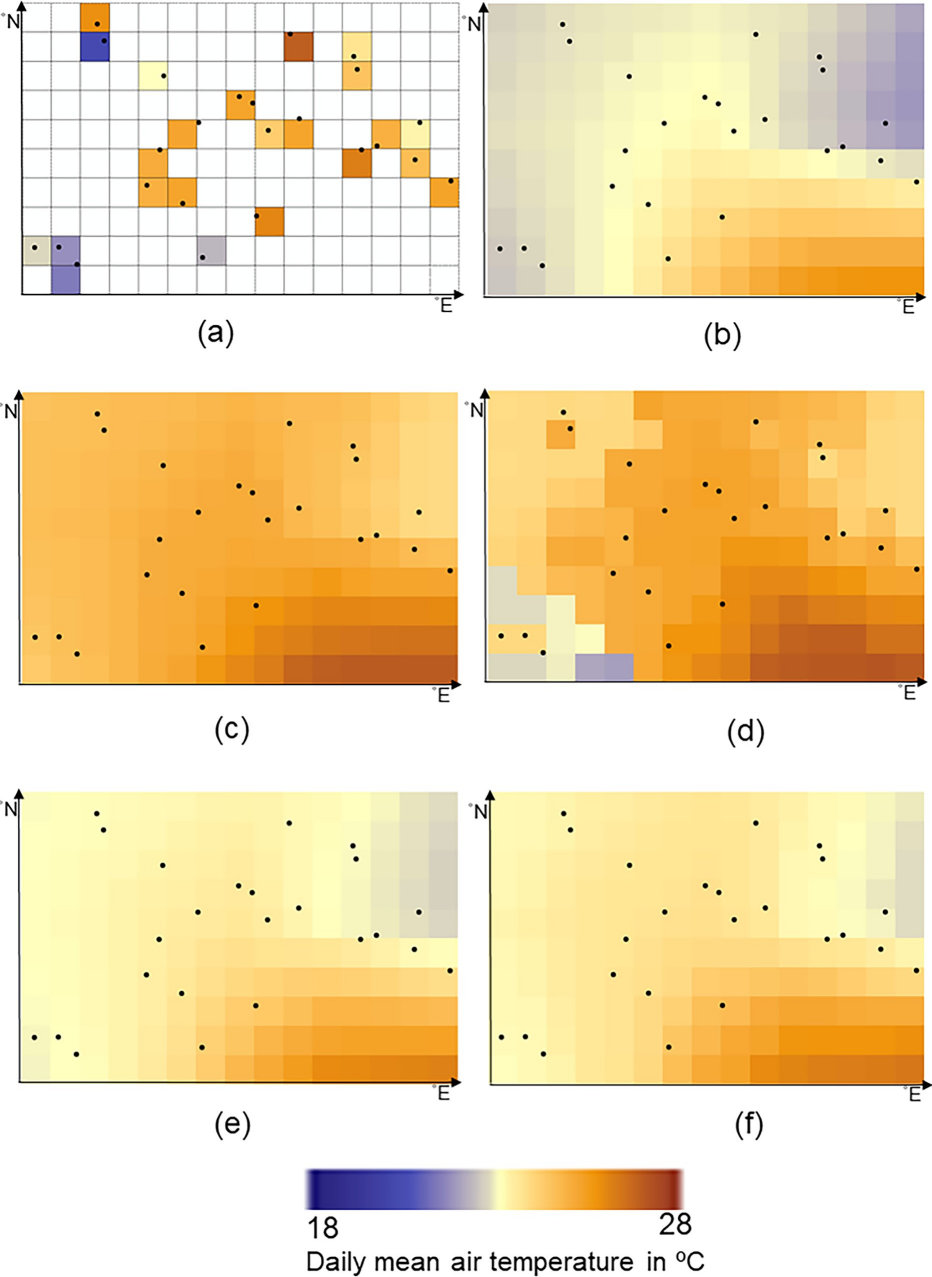
The mean air temperatures from a) weather stations, b) reanalysis data, and results of c) CP-I, d) CP-II, e) CE and f) CM, for all locations at June 1st 2014. For experimentation in this study, a sample subset of 10 × 15 grid cells of ECMWF dataset is selected at 0.125° lat/lon distances. The study area extends from 35.44° to 36.68° latitudes (N) and from 49.09° to 50.92° longitudes (E).

Two time series of the bias-corrected values obtained by CP-I and CP-II (Figs 6A and 5B) at the first station are compared with those of CE and CM (Fig 6C and 6D). The spatial mean absolute errors at this station for CP-II and CP-I were equal to 1.56°C and 1.66°C, whereas for CM and CE, they were equal to 2.72°C and 2.95°C, respectively. Bias-corrected values at June 1^st^ 2014 are shown in Fig 7. For CP-II and CP-I, the temporal mean absolute errors were equal to 2.17°C and 2.23°C at this day, whereas for CM and CE, they were equal to 2.41°C and 2.49°C, respectively.

**Fig 8:**
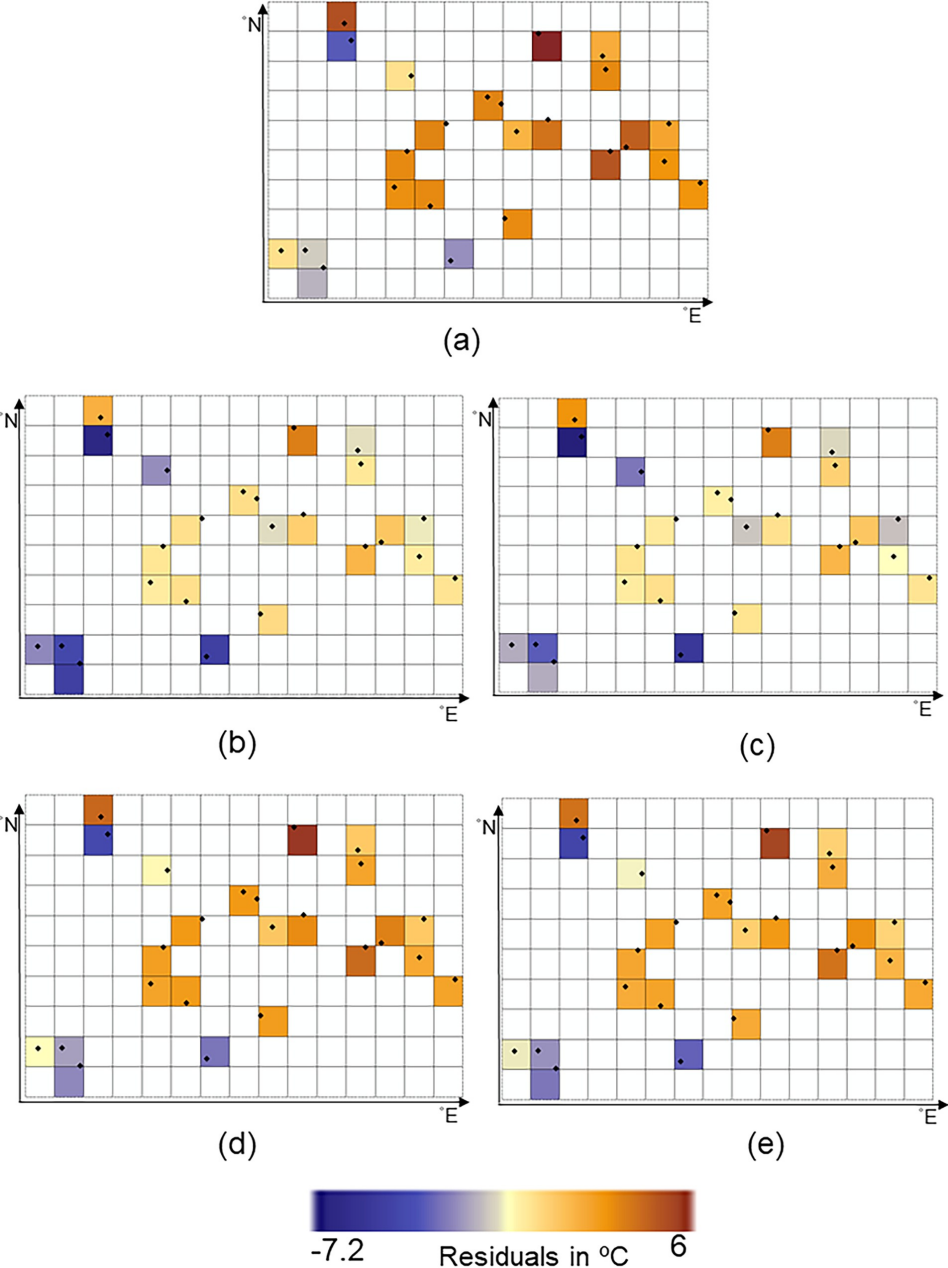
Bias (a) and prediction errors. Prediction errors are differences between the mean air temperatures from weather stations and the predictions obtained by b) CP-I, c) CP-II, d) CE and e) CM at June 1st 2014. For experimentation in this study, a sample subset of 10 × 15 grid cells of ECMWF dataset is selected at 0.125° lat/lon distances. The study area extends from 35.44° to 36.68° latitudes (N) and from 49.09° to 50.92° longitudes (E).

We note that CP-I fails to predict spatial variation and extremes in space (Fig 7C) but that CP-II is successful (Fig 7D) as compared to spatial variation of the measurements at this day (Fig 7A). Spatial variation of the bias-corrected values obtained by CP-I (Fig 7C), CE (Fig 7E) and CM (Fig 7F) is similar to spatial variation of the reanalysis air temperatures (Fig 7B). Spatial variation of the bias-corrected values obtained by CP-II differs from spatial variation of the reanalysis air temperatures (Fig 7B) because the optimal conditional probability obtained by this method changes in space. Bias and prediction errors at June 1^st^ 2014 are shown in Fig 8. The mean absolute bias is 2.84°C at this day, whereas the mean absolute prediction errors for CP-II and CP-I were equal to 1.13°C and 1.66°C, and for CE and CM to 2.46°C and 2.31°C, respectively.

As noted above, we applied a leave-*k*-out cross-validation where *k* indicates the number of the observations in 11 successive years at one day and one station. MAE obtained for two experiments (Table 4) shows that CP-II performed best, followed by CP-I, CM and CE. The MAE is slightly above 2°C for all methods whereas the average of absolute bias is 3.6°C. The horizontal distances, different height and differences in land cover between the location of a station and the grid cell center might affect the MAE. Investigating the CP-II including elevation, we noticed a large improvement in the results: the MAE for CP-II including elevation was equal to 1.92°C whereas for CP-II it was equal to 2.17°C (Table 4).

**Table 4:** Comparison of the bias correction methods for two experiments. The methods are applied on 24 weather stations in the first experiment whereas they are applied on a subset of ten stations in the second experiments. Total mean absolute error (MAE), spatial error scores (SES), temporal error scores (TES), spatial correlation scores (SCS), and temporal correlation scores (TCS), obtained by the conditional probabilities (CP-I, CP-II and CP-II including elevation), conditional expectation (CE) and conditional median (CM). The underlined values denote the best method. Only MAE is obtained for CP-II including elevation.

Method	MAE	SES	TES	SCS	TCS
	**Results of the 1**^**st**^ **experiment**				
**CP-I**	2.28	52	59	71	80
**CP-II**	2.17	55	34	86	120
**CP-II including elevation**	1.92	-	-	-	-
**CE**	2.45	71	116	54	49
**CM**	2.41	62	91	29	51
	$\mathrm{Results}\;\mathrm{of}\;\mathrm{the}\;2^{\mathrm{nd}}\;\mathrm{experiment}$				
**CP-I**	1.44	27	70	32	80
**CP-II**	1.36	19	47	37	102
**CE**	1.50	28	92	20	56
**CM**	1.50	26	91	11	62

We used SES and SCS to compare the presented methods based upon errors and correlations in time, i.e. 30 days in June (as shown in S1–S3 Figs). For the comparison in space, TES and TCS were used with *N =* 24 (as shown in S6–S8 Figs). Table 4 shows that CP-I resulted into the lowest errors in time whereas CP-II resulted into the lowest errors in space and highest correlations in space and time. The correlations *r*_*t*_ show that CP-II performed better in reproducing the spatial variation of the daily air temperatures in the study area (see S9A Fig). The correlations *r*_*t*_ obtained by CP-I, CE and CM are similar to the correlations between the reanalysis values and the measured values (cf. S9A Fig). This is as expected, because the predictor is the same for all locations in space. The correlations *r*_*s*_ denote that CP-I performed better in reproducing the temporal variation of the daily air temperatures in June (cf. S9B Fig).

Investigating the differences in quality of the measurements at the weather stations, we compared the spatial mean absolute prediction error (see Eq 8) with the spatial mean absolute bias. In this way, we assessed the performance of the bias correction methods at three types of the weather stations (cf. S10 Fig). This investigation showed that the predictions at two synoptic stations i.e. stations 6 and 19 are influenced by different sources of uncertainties in the measurements derived from three types of the weather stations. In addition, CP-II performed better than CE and CM.

The previous comparisons showed the performance of the methods based upon an individual criterion. To evaluate the performance based upon all criteria, we ranked the methods in each column of Table 4 where the lowest rank value denotes the best method. Table 5 shows the score of each method based upon the criteria mentioned in Table 4. We obtained an overall score using the sum of the scores. This overall score shows that CP-II reduced the bias with 63–68% for the full data set and with 69–74% on a homogeneous subarea whereas CP-I reduced the bias with 44–53% for the full data set and with 34–47% on a homogeneous subarea (Table 5, last column).

**Table 5:** Overall score based upon Table 3 for two experiments. The methods are applied on 24 weather stations in the first experiment whereas they are applied on a subset of ten stations in the second experiments. The scores are obtained for each method based upon each criterion i.e. each column of Table 3 where the lowest score denotes the best method. Overall score is the sum of the scores. The underlined values denote the best method.

Method	Score based on MAE	Score based on SES	Score based on TES	Score based on SCS	Score based on TCS	Overall score
	**Results of the 1**^**st**^ **experiment**					
**CP-I**	2	1	2	2	2	9
**CP-II**	1	2	1	1	1	6
**CE**	4	4	4	3	4	19
**CM**	3	3	3	4	3	16
	$\mathrm{Results}\;\mathrm{of}\;\mathrm{the}\;2^{\mathrm{nd}}\;\mathrm{experiment}$					
**CP-I**	2	2	2	2	2	10
**CP-II**	1	1	1	1	1	5
**CE**	4	4	4	3	4	19
**CM**	3	3	3	4	3	16

## Discussion

In this paper, we presented and evaluated two new bias correction methods for air temperature that take temporal and spatial variations into account. The CE and CM methods produce smooth maps, assuming spatial stationarity when estimating the dependence structures between the measured and the reanalysis weather data. We proposed to use different conditional probabilities minimizing the bias in space to improve spatial variation of the bias-corrected values. In addition, we described the dependence structure between the measured and the reanalysis weather data using the flexibility of selecting best fitting family among five copula families.

A Copula is a joint distribution function. Initially, the joint distribution is fitted to the data, and the goodness of fit is tested using statistical tests. Next, a predictor is selected to predict the variable of interest. The choice of the predictor is governed by the loss functions. This paper highlights the difference between estimation and prediction [32]. For instance, the mean and the median are predictors that minimize both the squared error loss and absolute error loss. These predictors produce smooth maps where spatial stationarity is assumed in estimating bivariate joint distributions. The predictors, CP-I and CP-II, were defined based upon varying conditional probabilities to improve spatial predictions. This flexibility is a practical advantage of implementing copulas when estimating distributions.

In our application, a bivariate copula was fitted to daily observations of the involved variables assuming spatial stationarity, and the bias correction was applied separately on each day. The results showed that the our methods performed better to correct time series of the air temperatures i.e. temporal variation of the daily air temperatures in June 2014. Therefore, a practical advantage of the new methods is that they are not any longer restricted to remove autocorrelation and heteroscedasticity in time series. A novel aspect is the potential and the use of the new methods for other copula-based methods such as interpolation and downscaling where the variable of interest needs to be predicted.

By means of the comparison of the methods based upon error scores and correlation scores, we demonstrated that CP-I performed best in time, whereas CP-II performed best in space. As the copulas are generally able to describe spatio-temporal dependences, the use of the spatio-temporal information in CP-II might help to improve its performance in time as well. We selected the number of neighbours based upon our experience. A more generally applicable sensitivity analysis is necessary to show the effects of the number of nearest neighbours on performance of CP-II.

We identified several routes for future research. First, we treated the measurements from weather stations as the benchmarks in the identification of bias and in the cross-validation. To address the uncertainty of the measurements and its impact on the results of the proposed methods, the proposed methods should be extended towards other datasets. In addition, further applications of the new copula-based methods in other case studies including simulation-based information should provide more insight on these methods. Second, we used the AIC to select the best fitting family. The bivariate Gaussian, Clayton, Gumbel and Frank families have a single parameter related to correlation, whereas the Student’s *t* family has one parameter for correlation and one parameter for the degrees of freedom. If the Bayesian Information Criteria (BIC) is chosen, the penalty for two parameter family, here Student’s *t* family, is larger than when using the AIC. We found that the best fitting families selected by AIC and BIC were the same for all days except for day 8 when Student’s *t* family was selected by the AIC and Frank family by the BIC. We realized though that the suitability of a copula also depends on the number of data used for fitting and the probabilistic nature of the bias. Further cross validations need to be carried out using random samples of the measurements to choose the copula family. Third, spatially varying conditional probabilities needs to be further applied in other methods e.g. Bayes' classifier and possibly in a machine learning environment. Fourth, to extend the current study, the use of multivariate copula describing the dependence between more variables e.g. air temperature, elevation and land cover might help to improve the performance of the presented methods. The bivariate case of the proposed methods in this paper is useful if such a covariate is unavailable. Finally, a comparison to other bias correction methods e.g. quantile mapping might be included in further studies.

## Conclusions

We proposed to use conditional probabilities to correct for bias in the gridded reanalysis weather data provided by ECMWF as compared to the measurements from weather stations taken as the benchmarks. Cross-validation results and correlation scores showed that the new methods perform better than commonly applied methods and are able to account for spatial and temporal variation of air temperatures at unvisited locations.

## Supporting information

S1 Appendix Properties of the conditional expectation. (DOCX)

S2 Appendix Conditional copula density. (DOCX)

S3 Appendix Evaluating the stationarity assumption. (DOCX)

S1 Table 24 weather stations in the study area. The quality of measurements and number of missing values differ at each station.
(DOCX)

S2 Table The values of co-correlogram and best fitting family at five spatial lags. Kendall’s τ correlations are obtained using the measured and reanalysis values on each day in June from 24 weather stations between 2004 to 2014. The copula families are: N = Gaussian, T = Student’s *t*, C = Clayton, G = Gumbel and F = Frank.
(DOCX)

S1 Fig (TIF)

S2 Fig (TIF)

S3 Fig The vertical axis is daily mean air temperature in °C. The number on each graph denotes the weather station number. Time series of the measurements from weather stations, reanalysis data and bias corrected values obtained by the bias correction methods at each station in June 2014.
(TIF)

S4 Fig Variation of the mean air temperature on 1st day of June 2014 comparing with variation of the elevation in the study area. The mean air temperature in °C are derived from the synoptic and climatology type 1 weather stations.
(TIF)

S5 Fig Influence of the choice of the increment value (IV) on a) the optimal conditional probability in CP-I and b) the mean absolute prediction errors. Three IVs 0.1, 0.01 and 0.001 are chosen.
(TIF)

S6 Fig (TIF)

S7 Fig (TIF)

S8 Fig The daily mean air temperatures from weather stations, reanalysis data and bias corrected values obtained by the bias correction methods for all locations on each day in June 2014. The number on each graph denotes the day in June 2014.
(TIF)

S9 Fig The correlation coefficients r: a) in space on each day in June 2014, b) in time at each weather station. The numbers on the figures denote correlations.
(TIF)

S10 Fig Comparing mean absolute prediction error with mean absolute bias at three types of the weather stations. The vertical axis is error/bias in °C. The synoptic stations are supposed to provide more precise measurements.
(TIF)

S11 Fig p values of the regression parameters in trend analysis obtained by *F* test. Based upon its results, spatial stationarity is assumed in estimating the marginal distribution.
(TIF)

S12 Fig The values of correlogram at five spatial lags. The vertical axis is Kendall’s τ correlations obtained using the measurements on each day in June between 2004 to 2014. The horizontal axis is spatial lags in meter.
(TIF)
