# Supplementary material for: The use of bivariate copulas for bias correction of reanalysis air temperature data
Source: PLoS One. 2019 May 8;14(5):e0216059. doi: 10.1371/journal.pone.0216059 (PMC6505955; doi:10.1371/journal.pone.0216059)
Supplement: S1 Appendix — (DOCX) [file pone.0216059.s001.docx]

# S1 Appendix. Properties of the conditional expectation

The conditional expectation is defined as:

$\hat{x}=E\left[ X | Y=y \right]=\int_{x} x\cdot f\left( x | y \right)dx=\int_{0}^{1} F_{X}^{-1}\left( u \right)\times c(u|V=v)du$.

Let ($x_{1}, y_{1}$), ($x_{2}, y_{2}$), …, ($x_{n}, y_{n}$) be a set of paired observations for variables $X$ and $Y$. If $x_{1}> x_{2}$ and $y_{1}> y_{2}$ or if $x_{1}< x_{2}$ and $y_{1}< y_{2}$, the pairs are called concordant, whereas if $x_{1}> x_{2}$ and $y_{1}< y_{2}$ or if $x_{1}< x_{2}$ and $y_{1}> y_{2}$ they are discordant. When the number of concordant pairs $n_{c}$ is more than the number of discordant pairs $n_{d}$, the dependence between $X$ and $Y$ is positive, whereas when $n_{c}<n_{d}$, the dependence is negative [1]. Hence, if a bivariate copula represents a positive correlation, the conditional expectation is an increasing function of the conditioning variable, i.e., if $y_{1}> y_{2}$, then $E[X| y_{1}]>E[X| y_{2}]$ [2]. If a bivariate copula represents a negative correlation, the conditional expectation is a decreasing function, therefore, if $y_{1}< y_{2}$ then $E[X| y_{1}]<E[X| y_{2}]$.

The last equality in the conditional expectation is explained based upon Sklar’s theorem as:

$$f\left( x | y \right)=\frac{f\left( x,y \right)}{f_{Y}\left( y \right)}=\frac{c\left( u,v \right)\times f_{X}\left( x \right)\times f_{Y}\left( y \right)}{f_{Y}\left( y \right)}=c\left( u|v \right)\times f_{X}\left( x \right)$$

$$=c\left( u|v \right)\times\frac{\partial F_{X}\left( x \right)}{\partial x}=c\left( u|v \right)\times\frac{\partial u}{\partial x}$$

The conditional density functions can be extended to higher dimensions.

# Reference

1. Nelsen RB. An Introduction to Copulas. United States of America: Springer; 2006. 276 p.

2. Dodds PG, Huijsmans CB, DePagter B. Characterizations of Conditional Expectation-Type operators. Pacific Journal of Mathematics 1990;141(1).
