# Supplementary material for: The use of bivariate copulas for bias correction of reanalysis air temperature data
Source: PLoS One. 2019 May 8;14(5):e0216059. doi: 10.1371/journal.pone.0216059 (PMC6505955; doi:10.1371/journal.pone.0216059)
Supplement: S2 Appendix — (DOCX) [file pone.0216059.s002.docx]

# S2 Appendix. Conditional copula density

We denote the conditional distribution function for $U$ given $v$ [1] as:

$$C\left( u|v \right)=P\left[ U\leq u | V=v \right].$$

In the case of constructing bivariate copulas, the conditional density $c\left( u|v \right)$ is equal to the joint density $c\left( u,v \right).$

**Proof:** In copulas, marginals $\left( U,V \right)$are uniformly distributed on [0,1] i.e. *f(u)=f(v)=*1, *F(u)=u* and *F(v)=v*, where *f* and *F* are density and cumulative distribution functions, respectively [2]. The conditional density is given as [1]:

$c\left( u|v \right)=\frac{c\left( u,v \right)}{f\left( v \right)}$, $f\left( v \right)=1$.

# Reference

1. Nelsen RB. An Introduction to Copulas. United States of America: Springer; 2006. 276 p.

2. Kuipers L, Niederreiter H. Uniform Distribution of Sequences: Dover Publications; 2012.
