# Supplementary material for: The use of bivariate copulas for bias correction of reanalysis air temperature data
Source: PLoS One. 2019 May 8;14(5):e0216059. doi: 10.1371/journal.pone.0216059 (PMC6505955; doi:10.1371/journal.pone.0216059)
Supplement: S3 Appendix — (DOCX) [file pone.0216059.s003.docx]

# S3 Appendix. Evaluating the stationarity assumption

To evaluate the second order spatial stationarity assumption in estimating marginal distribution of daily air temperature, we used two methods: linear regression and auto-correlation function. The null hypothesis $H_{0}$ and alternative hypothesis $H_{1}$ to test for second order stationarity assumption are then defined as:

$H_{0}: E[Z_{s}]=\mu$,

$$H_{1}: E\left[ Z_{s} \right]=\beta_{0}+\beta_{1}.x_{s}+\beta_{2}.y_{s},$$

where $Z_{s}$ is the variable of interest at location $s$, *E[]* denotes the expectation, $x_{s}$ and $y_{s}$ are the *x* and *y* coordinates of location $s$ and the $\beta_{j}, j=0, 1, 2$ are regression parameters. We obtained the parameters and their *p* values using a linear model and *F* test [1]. We found that the values of regression coefficients are not significantly different from zero and their *p* values of *F* test are above 0.05 and 0.01 at all days (cf. S11 Fig). The auto-correlation function, i.e. correlogram, describes dependences in space based upon the correlation per each spatial lag [2]. The values of correlogram at five spatial lags are obtained from the measured values on each day in June between 2004 and 2014 (cf. S12 Fig). It is immediate that the correlations are decreasing by the separating distance. These results and the limited effects of including non-stationarity make the assumption of spatial stationarity a reasonable one.

We assess the second order spatial stationarity assumption in estimating copula using co-correlation function. Co-correlation function, i.e. the co-correlogram, is an extension of the correlogram for two or more random fields in space. The values of the co-correlogram and the best fitting family at five spatial lags are obtained from measured and reanalysis values on each day in June between 2004 and 2014 (cf. S2 Table). The results show that the best fitting families and the correlations differ slightly at different spatial lags. Therefore, we conclude that the spatial stationarity is a reasonable assumption in estimating copula and point to further application of co-correlogram in lag based bias correction methods.

# Reference

1. Chambers J, Hastie TDP. Statistical Models in S. K. M, V. M, editors: Compstat. Physica-Verlag HD.; 1990.

2. Oden NL. Assessing the Significance of a Spatial Correlogram. Geographical analysis. 1984;16(1):1–16. doi: 10.1111/j.1538-4632.1984.tb00796.x.
