# Supplementary material for: The use of bivariate copulas for bias correction of reanalysis air temperature data
Source: PLoS One. 2019 May 8;14(5):e0216059. doi: 10.1371/journal.pone.0216059 (PMC6505955; doi:10.1371/journal.pone.0216059)
Supplement: S1 Table — The quality of measurements and number of missing values differ at each station. (DOCX) [file pone.0216059.s004.docx]

**S1 Table. 24 weather stations in the study area. The quality of measurements and number of missing values differ at each station.**

| Station ID | Station name | Latitudeº | Longitudeº | Elevation  (m) | Type | Air temperature measurements |
| --- | --- | --- | --- | --- | --- | --- |
| 1 | Abeyk | 36.05 | 50.52 | 1278 | Synoptic | 3 hourly |
| 2 | Magsal | 36.13 | 50.12 | 1205 | Climatology type1 | 6 hourly |
| 3 | Nirougah | 36.18 | 50.25 | 1299 | Climatology type1 | 6 hourly |
| 4 | Qazvin | 36.25 | 50.05 | 1280 | Synoptic | 3 hourly |
| 5 | Takestan | 36.05 | 49.65 | 1326 | Synoptic | 3 hourly |
| 6 | Avaj | 35.63 | 49.22 | 1888 | Synoptic | 3 hourly |
| 7 | Baghkelaye | 36.39 | 50.50 | 1256 | Climatology type2 | min and max |
| 8 | Baghkosar | 36.07 | 50.58 | 1541 | Climatology type1 | 6 hourly |
| 9 | Bouinzahra | 35.77 | 50.07 | 1213 | Synoptic | 3 hourly |
| 10 | Bourmanak | 36.59 | 49.38 | 578 | Climatology type2 | min and max |
| 11 | Camp | 36.28 | 49.99 | 1311 | Climatology type2 | min and max |
| 12 | Danesfahan | 35.82 | 49.75 | 1303 | Climatology type2 | min and max |
| 13 | Dolatabad | 36.17 | 49.82 | 1249 | Climatology type2 | min and max |
| 14 | Estalaj | 35.56 | 49.29 | 2340 | Climatology type2 | min and max |
| 15 | Hajiarab | 35.59 | 49.84 | 1707 | Climatology type2 | min and max |
| 16 | Hashtgerd | 36.01 | 50.75 | 1601 | Synoptic | 3 hourly |
| 17 | Jahanabad | 35.90 | 49.60 | 1372 | Climatology type2 | min and max |
| 18 | Karaj | 35.92 | 50.90 | 1657 | Synoptic | 3 hourly |
| 19 | Kouhin | 36.37 | 49.67 | 1498 | Synoptic | 3 hourly |
| 20 | Moalem | 36.45 | 50.48 | 1569 | Synoptic | 3 hourly |
| 21 | Niarak | 36.52 | 49.41 | 1184 | Climatology type2 | min and max |
| 22 | Qouzlo | 35.63 | 49.11 | 2061 | Climatology type2 | min and max |
| 23 | Razmiankia | 36.55 | 50.21 | 1010 | Synoptic | 3 hourly |
| 24 | Taleghan | 36.17 | 50.77 | 1827 | Synoptic | 3 hourly |
