# Supplementary material for: The use of bivariate copulas for bias correction of reanalysis air temperature data
Source: PLoS One. 2019 May 8;14(5):e0216059. doi: 10.1371/journal.pone.0216059 (PMC6505955; doi:10.1371/journal.pone.0216059)
Supplement: S2 Table — The copula families are: N = Gaussian, T = Student’s t, C = Clayton, G = Gumbel and F = Frank. (DOCX) [file pone.0216059.s005.docx]

**S2 Table. The values of co-correlogram and best fitting family at five spatial lags. Kendall’s τ correlations are obtained using the measured and reanalysis values on each day in June from 24 weather stations between 2004 to 2014. The copula families are: N=Gaussian, T=Student’s *t*, C=Clayton, G=Gumbel and F=Frank.**

|  | Best fitted family | | | | | Kendall’s τ correlation | | | | | |
| --- | --- | --- | --- | --- | --- | --- | --- | --- | --- | --- | --- |
| Day | 1 | 2 | 3 | 4 | 5 | 1 | 2 | 3 | 4 | 5 |  |
| 1 | G | G | G | G | G | 0.45 | 0.45 | 0.46 | 0.43 | 0.43 |  |
| 2 | N | N | N | N | N | 0.35 | 0.37 | 0.37 | 0.34 | 0.34 |  |
| 3 | N | G | G | G | G | 0.28 | 0.31 | 0.31 | 0.30 | 0.28 |  |
| 4 | N | G | G | G | G | 0.34 | 0.31 | 0.33 | 0.29 | 0.29 |  |
| 5 | N | T | T | T | T | 0.35 | 0.34 | 0.37 | 0.31 | 0.32 |  |
| 6 | F | F | F | F | F | 0.36 | 0.34 | 0.32 | 0.29 | 0.30 |  |
| 7 | G | N | G | G | G | 0.38 | 0.38 | 0.38 | 0.35 | 0.35 |  |
| 8 | G | G | G | T | T | 0.42 | 0.42 | 0.38 | 0.38 | 0.37 |  |
| 9 | N | G | G | G | F | 0.46 | 0.46 | 0.44 | 0.44 | 0.42 |  |
| 10 | G | G | G | G | G | 0.37 | 0.39 | 0.40 | 0.35 | 0.38 |  |
| 11 | G | G | G | G | G | 0.27 | 0.28 | 0.29 | 0.27 | 0.27 |  |
| 12 | N | N | N | N | N | 0.40 | 0.40 | 0.39 | 0.39 | 0.39 |  |
| 13 | N | N | T | N | T | 0.36 | 0.37 | 0.39 | 0.35 | 0.35 |  |
| 14 | N | N | N | N | N | 0.33 | 0.38 | 0.38 | 0.35 | 0.35 |  |
| 15 | N | G | G | G | G | 0.35 | 0.38 | 0.38 | 0.35 | 0.35 |  |
| 16 | N | G | G | G | G | 0.38 | 0.37 | 0.35 | 0.35 | 0.34 |  |
| 17 | N | G | G | G | G | 0.33 | 0.38 | 0.37 | 0.34 | 0.33 |  |
| 18 | F | F | F | F | F | 0.25 | 0.31 | 0.30 | 0.30 | 0.28 |  |
| 19 | F | F | F | F | F | 0.46 | 0.48 | 0.49 | 0.45 | 0.45 |  |
| 20 | G | G | G | G | G | 0.50 | 0.50 | 0.50 | 0.47 | 0.49 |  |
| 21 | F | G | G | G | G | 0.47 | 0.45 | 0.47 | 0.44 | 0.45 |  |
| 22 | G | G | G | G | G | 0.38 | 0.35 | 0.36 | 0.34 | 0.33 |  |
| 23 | F | G | G | G | F | 0.31 | 0.33 | 0.37 | 0.31 | 0.33 |  |
| 24 | G | G | G | G | G | 0.34 | 0.30 | 0.33 | 0.28 | 0.30 |  |
| 25 | G | G | G | G | G | 0.19 | 0.25 | 0.30 | 0.20 | 0.22 |  |
| 26 | G | G | G | G | G | 0.23 | 0.31 | 0.34 | 0.29 | 0.29 |  |
| 27 | G | N | N | N | N | 0.35 | 0.37 | 0.43 | 0.34 | 0.36 |  |
| 28 | F | F | F | F | F | 0.32 | 0.35 | 0.37 | 0.32 | 0.32 |  |
| 29 | N | F | F | F | F | 0.34 | 0.34 | 0.35 | 0.31 | 0.32 |  |
| 30 | G | G | G | G | G | 0.32 | 0.31 | 0.32 | 0.29 | 0.31 |  |
