# Supplementary figures and images for: The use of bivariate copulas for bias correction of reanalysis air temperature data
Source: PLoS One. 2019 May 8;14(5):e0216059. doi: 10.1371/journal.pone.0216059 (PMC6505955; doi:10.1371/journal.pone.0216059)

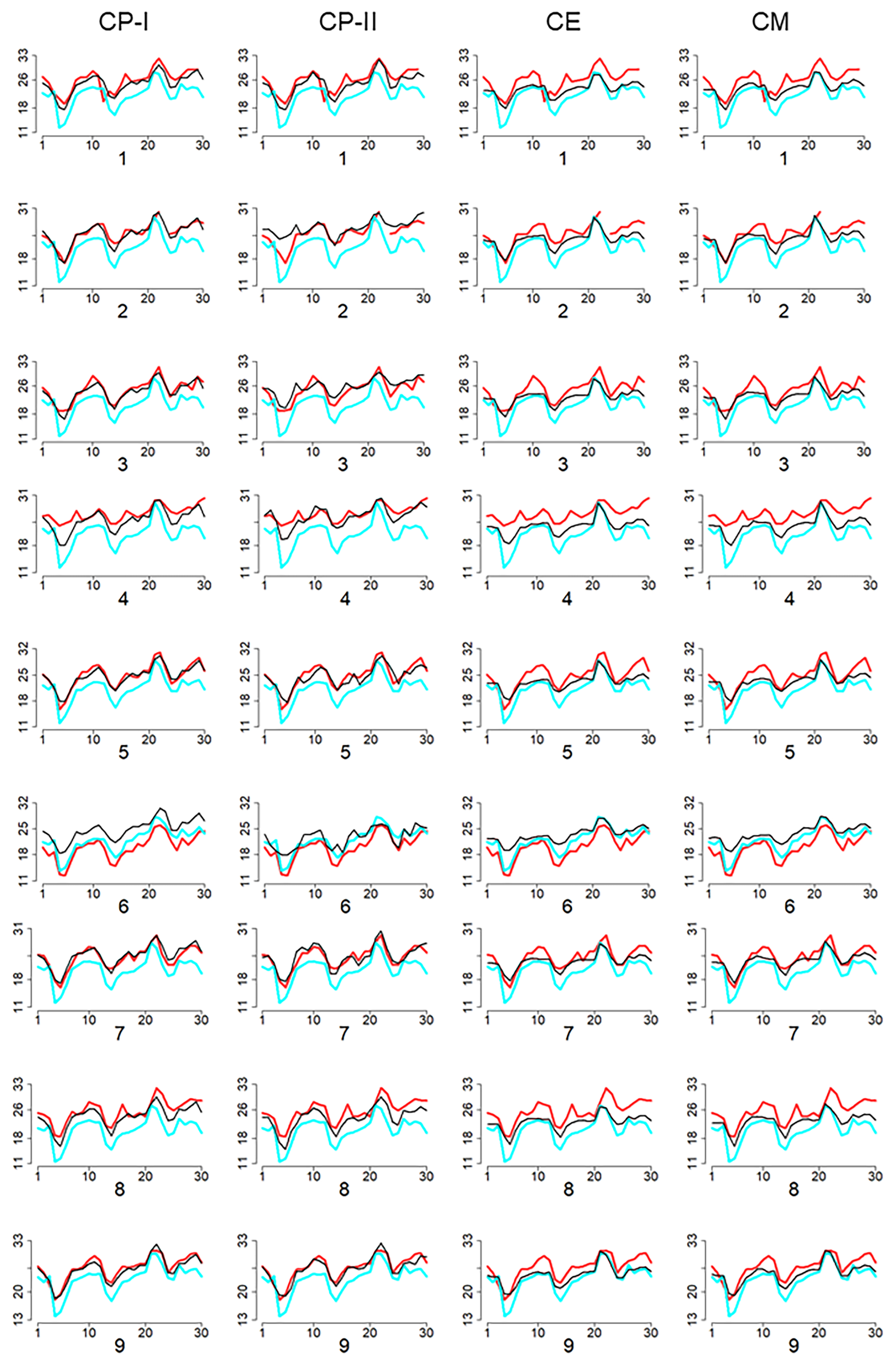

Supplement: S1 Fig — (TIF) [file pone.0216059.s006.tif]

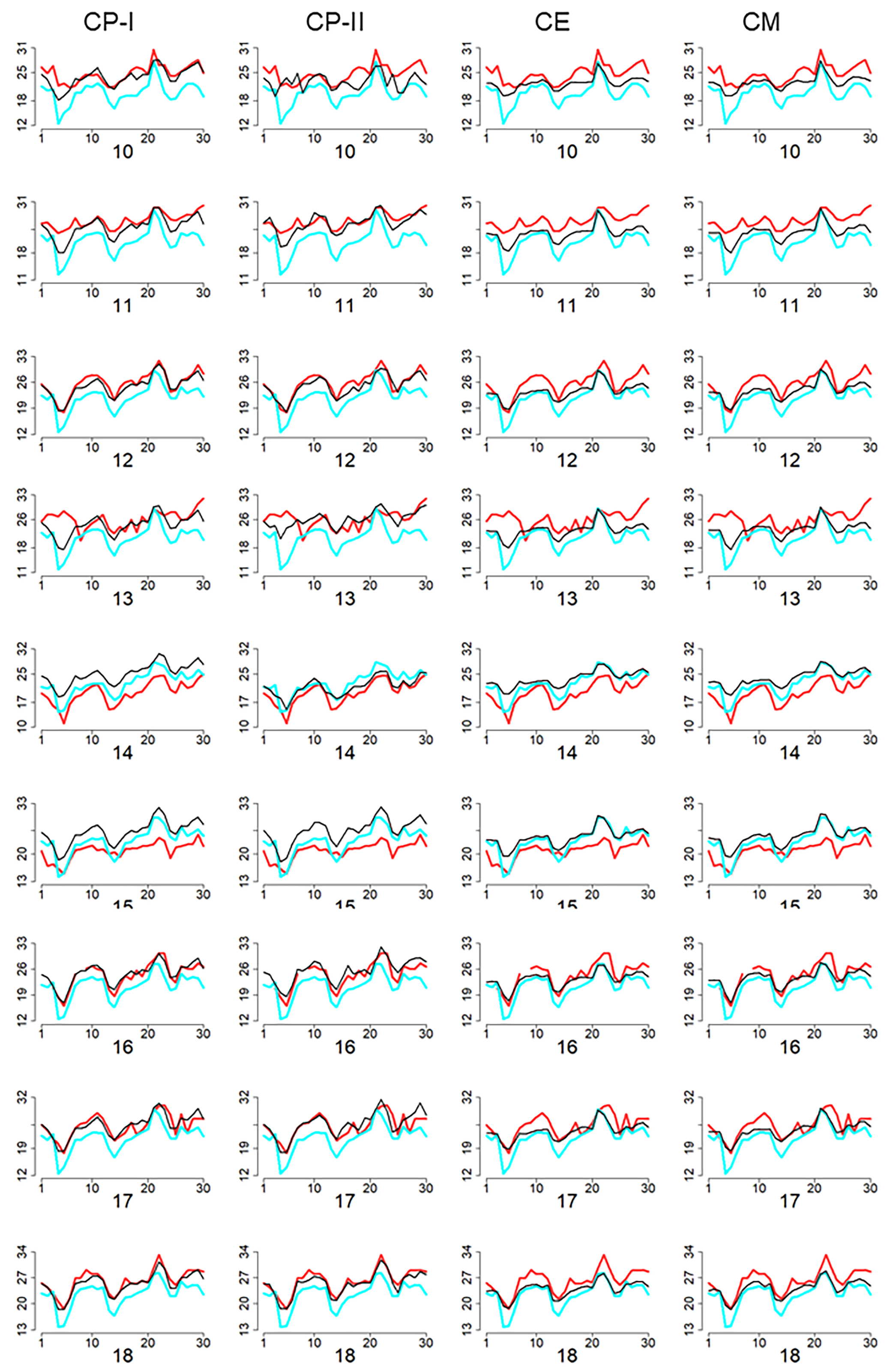

Supplement: S2 Fig — (TIF) [file pone.0216059.s007.tif]

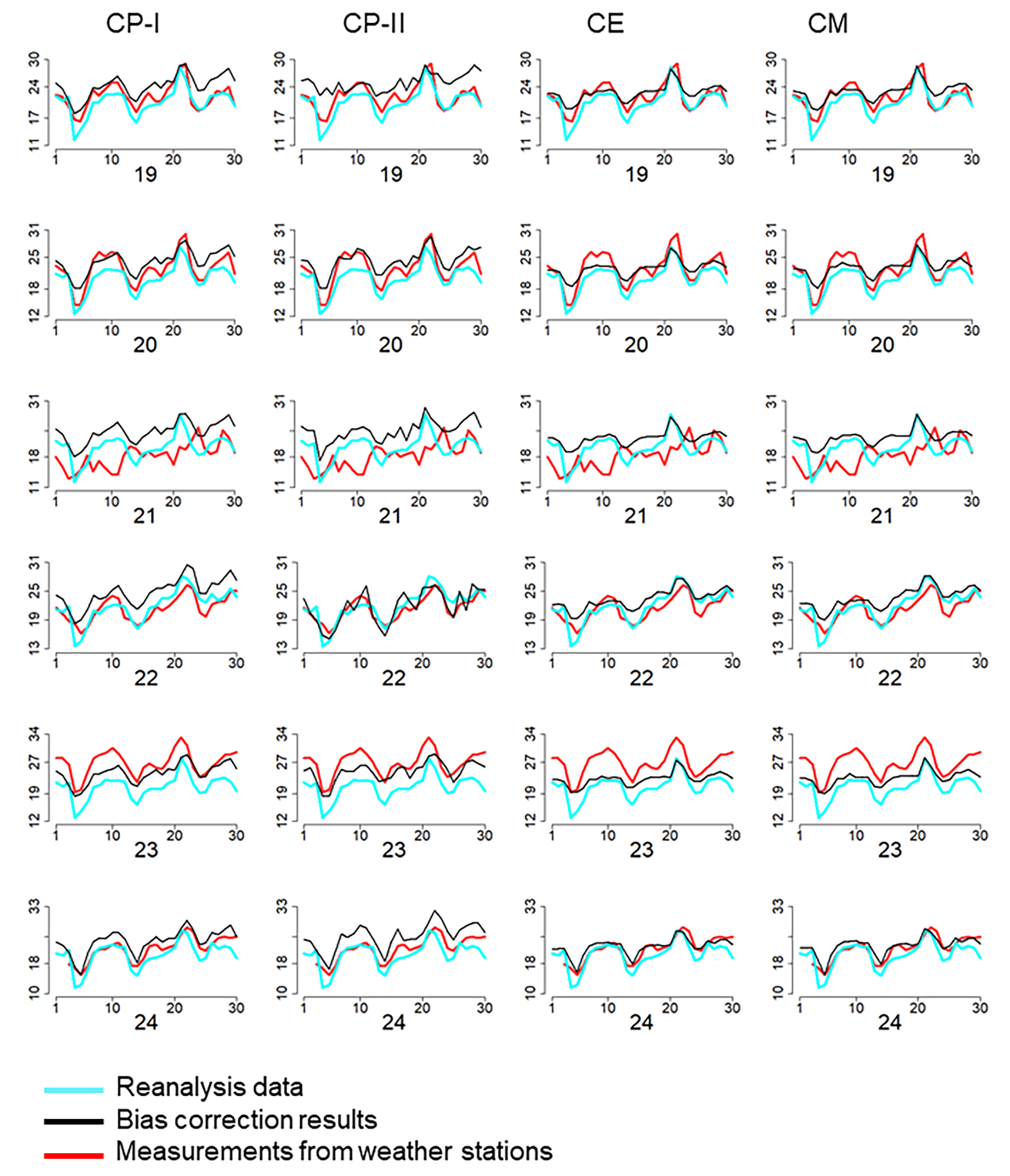

Supplement: S3 Fig — Time series of the measurements from weather stations, reanalysis data and bias corrected values obtained by the bias correction methods at each station in June 2014. (TIF) [file pone.0216059.s008.tif]

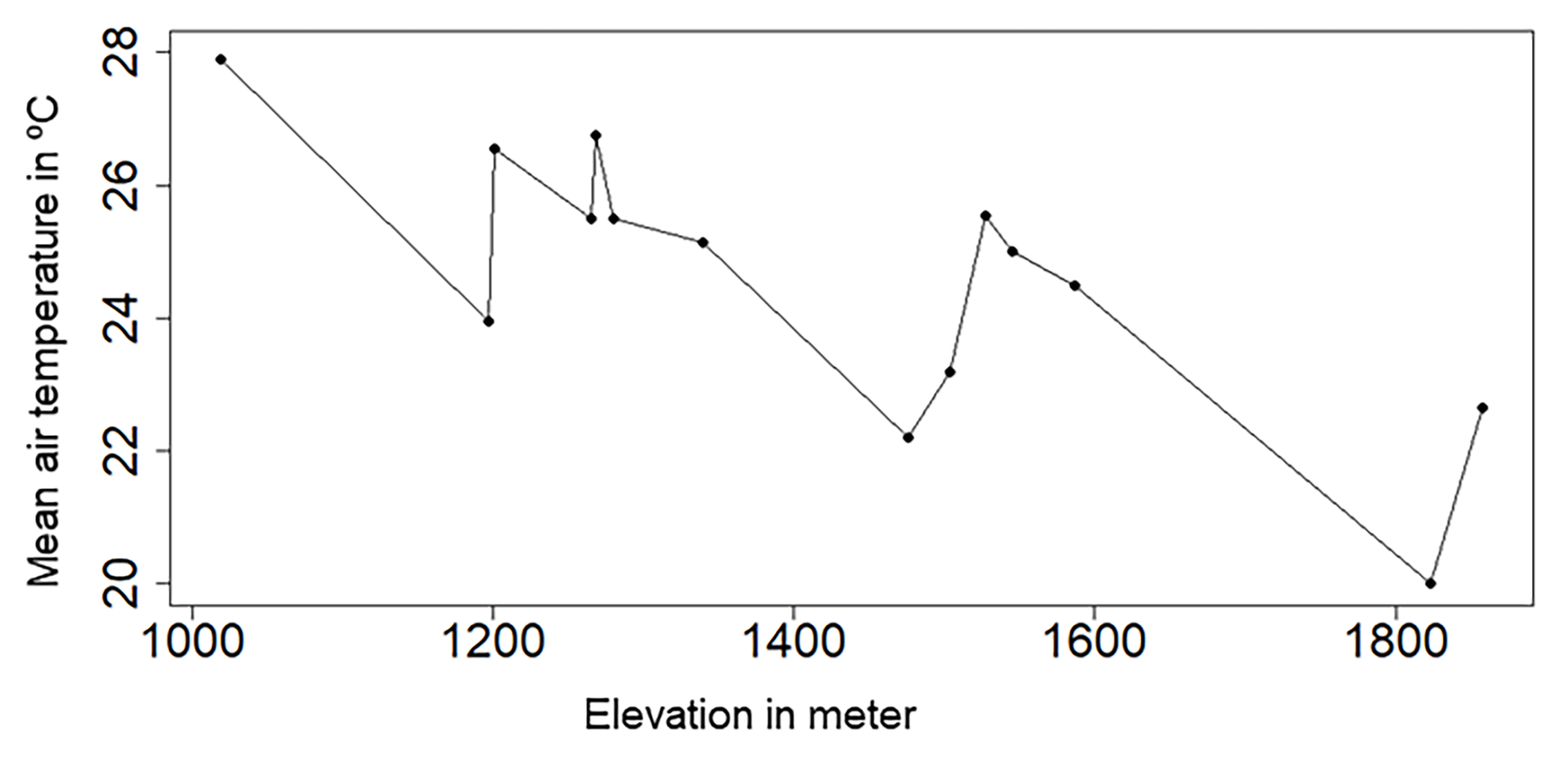

Supplement: S4 Fig — The mean air temperature in °C are derived from the synoptic and climatology type 1 weather stations. (TIF) [file pone.0216059.s009.tif]

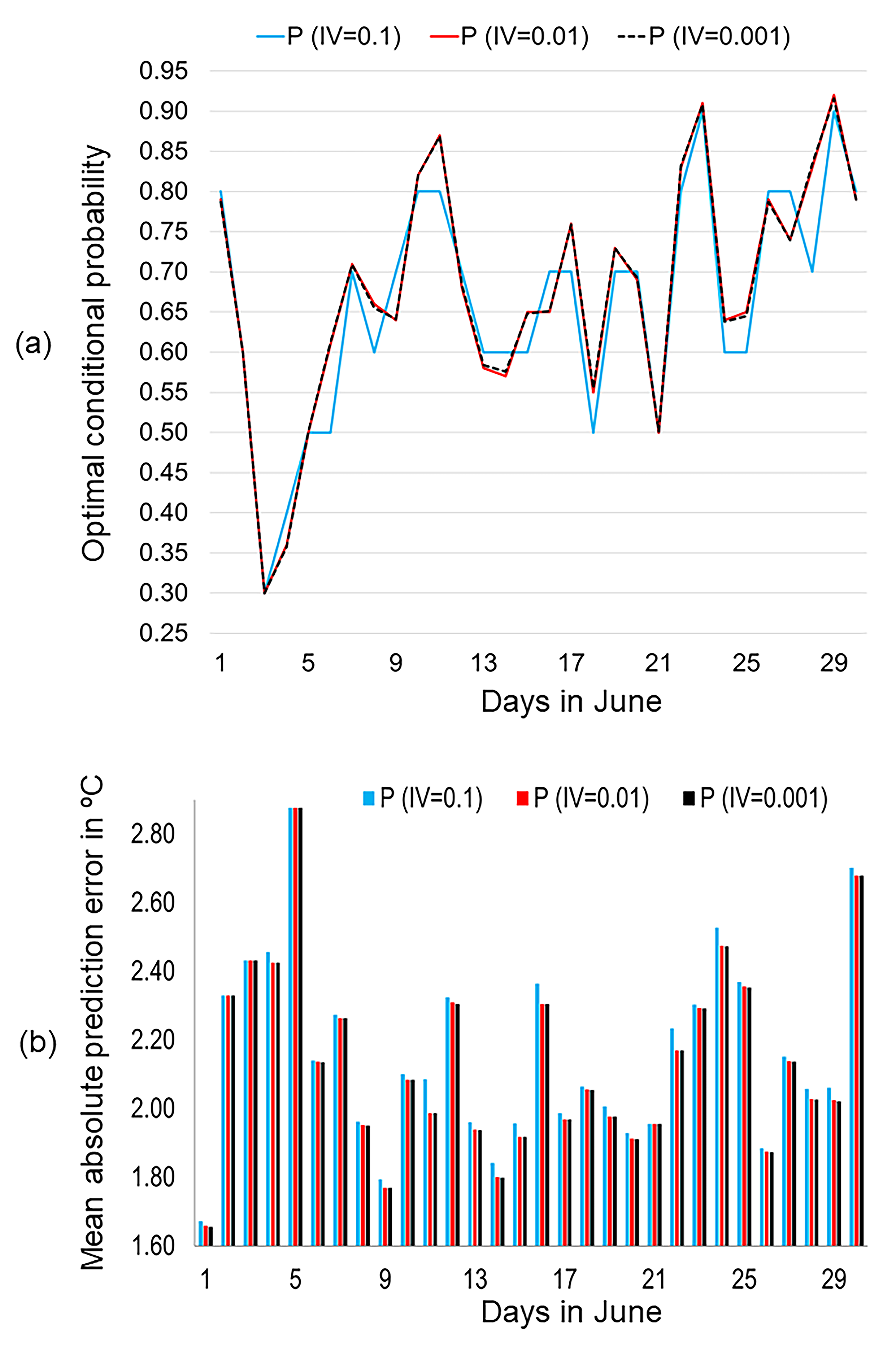

Supplement: S5 Fig — Influence of the choice of the increment value (IV) on a) the optimal conditional probability in CP-I and b) the mean absolute prediction errors. Three IVs 0.1, 0.01 and 0.001 are chosen. (TIF) [file pone.0216059.s010.tif]

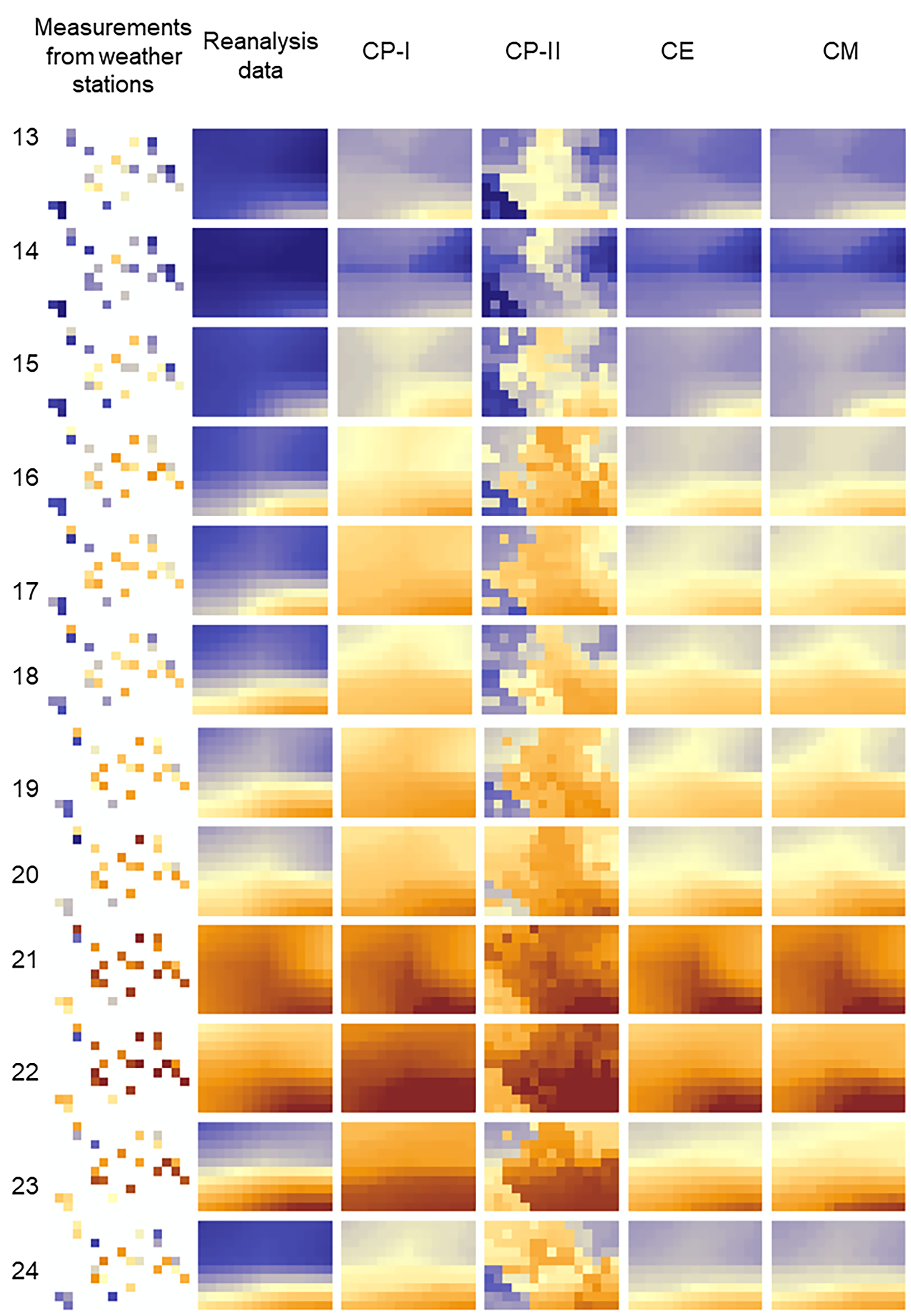

Supplement: S6 Fig — (TIF) [file pone.0216059.s011.tif]

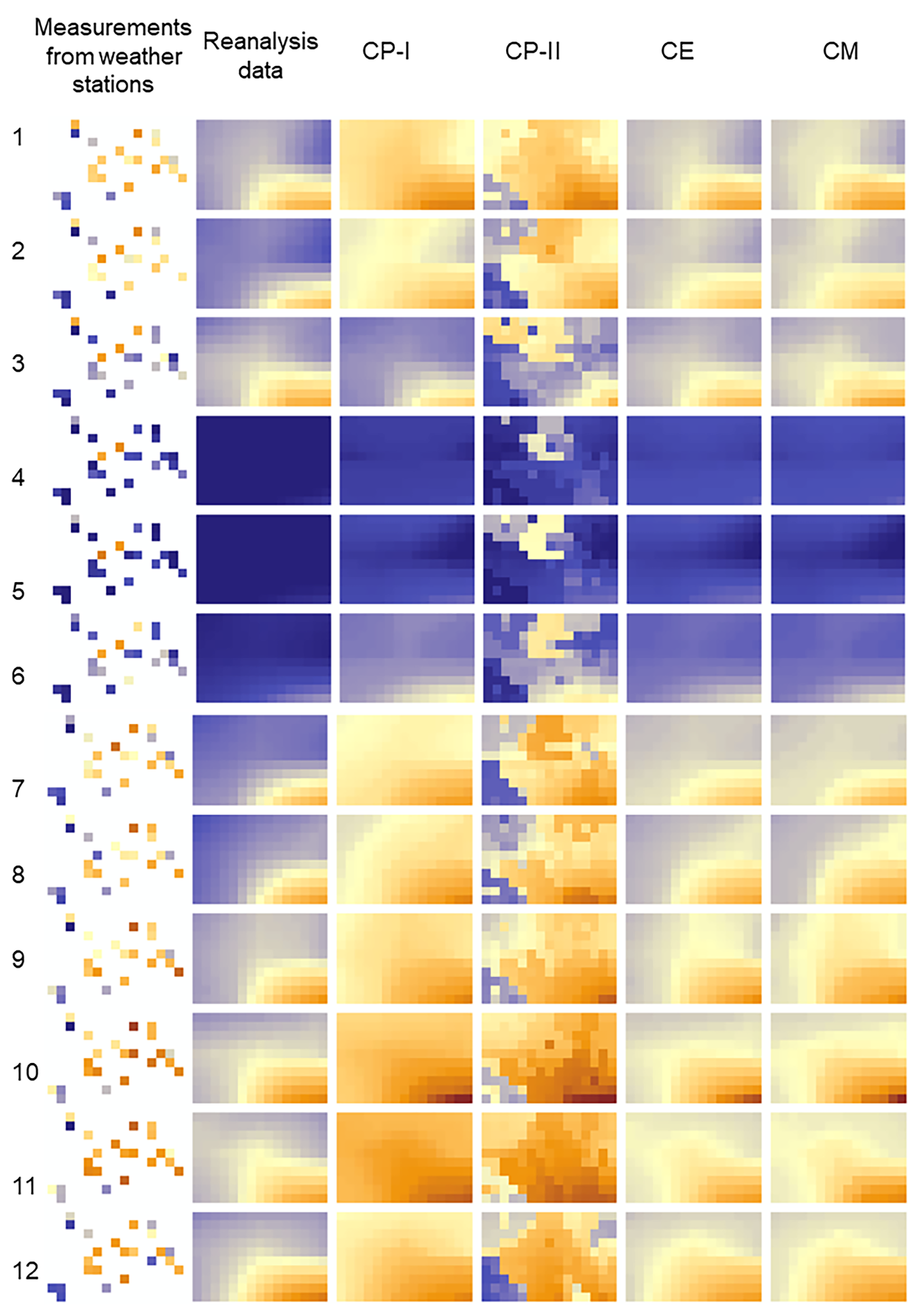

Supplement: S7 Fig — (TIF) [file pone.0216059.s012.tif]

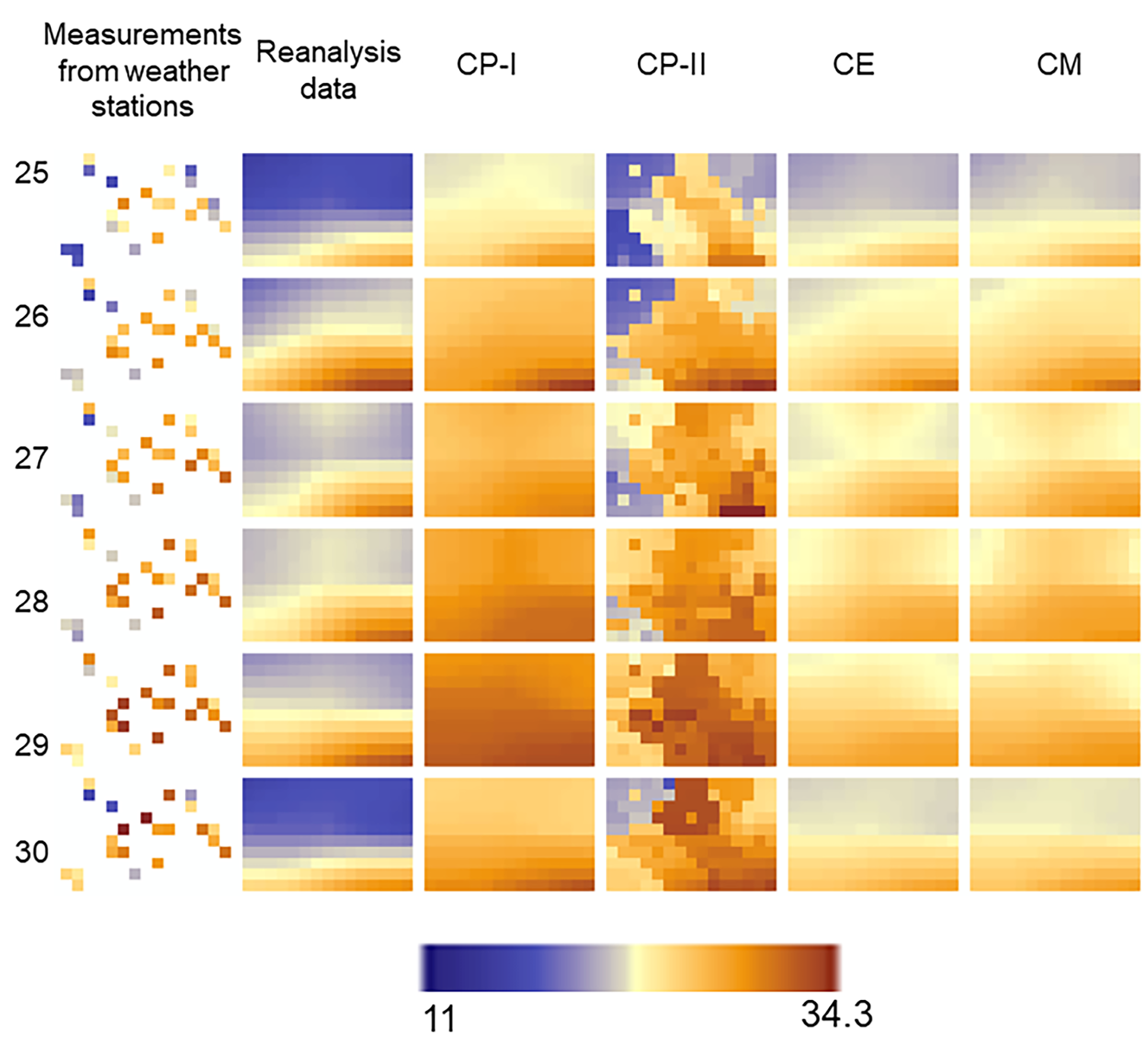

Supplement: S8 Fig — The number on each graph denotes the day in June 2014. (TIF) [file pone.0216059.s013.tif]

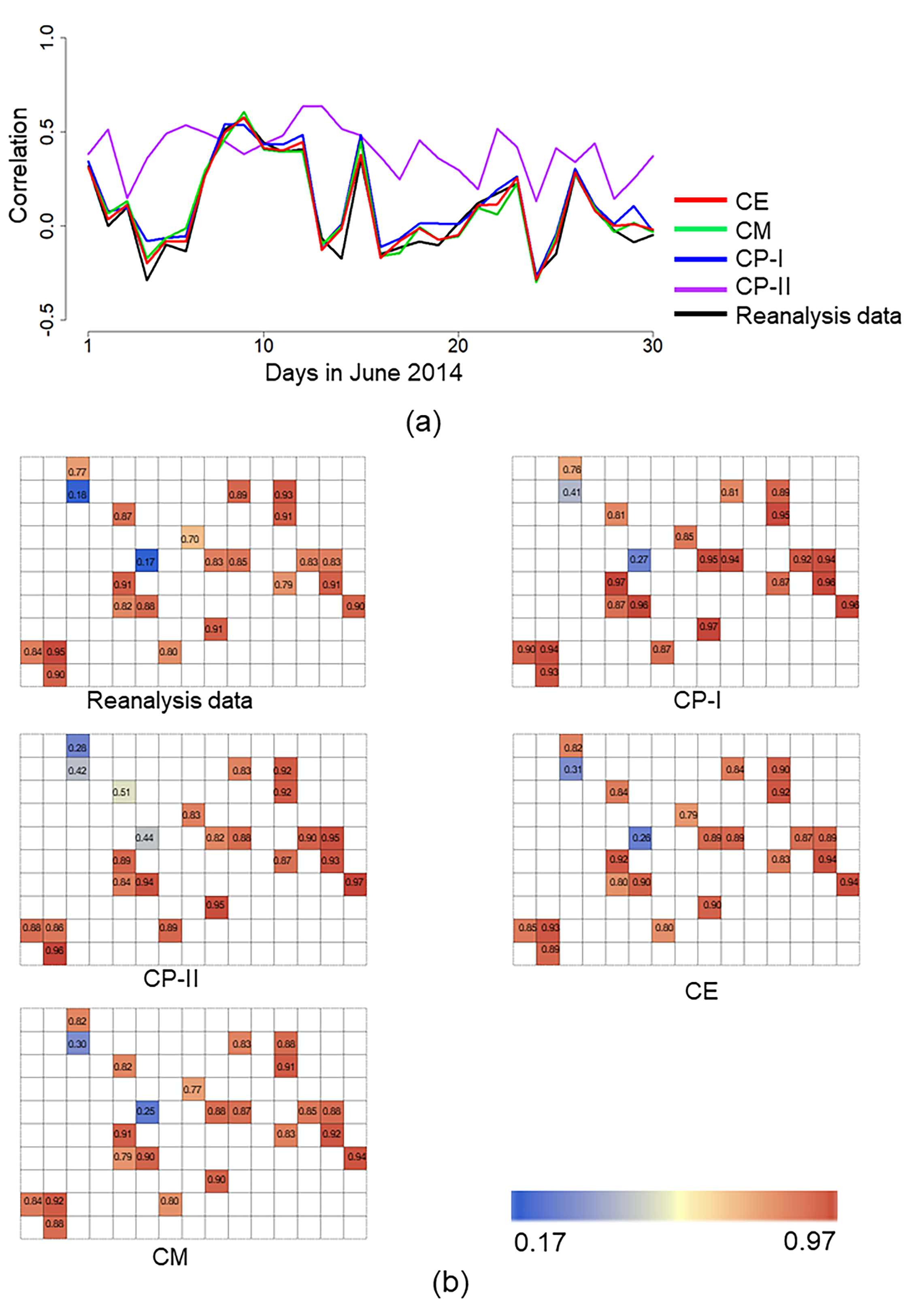

Supplement: S9 Fig — The correlation coefficients r: a) in space on each day in June 2014, b) in time at each weather station. The numbers on the figures denote correlations. (TIF) [file pone.0216059.s014.tif]

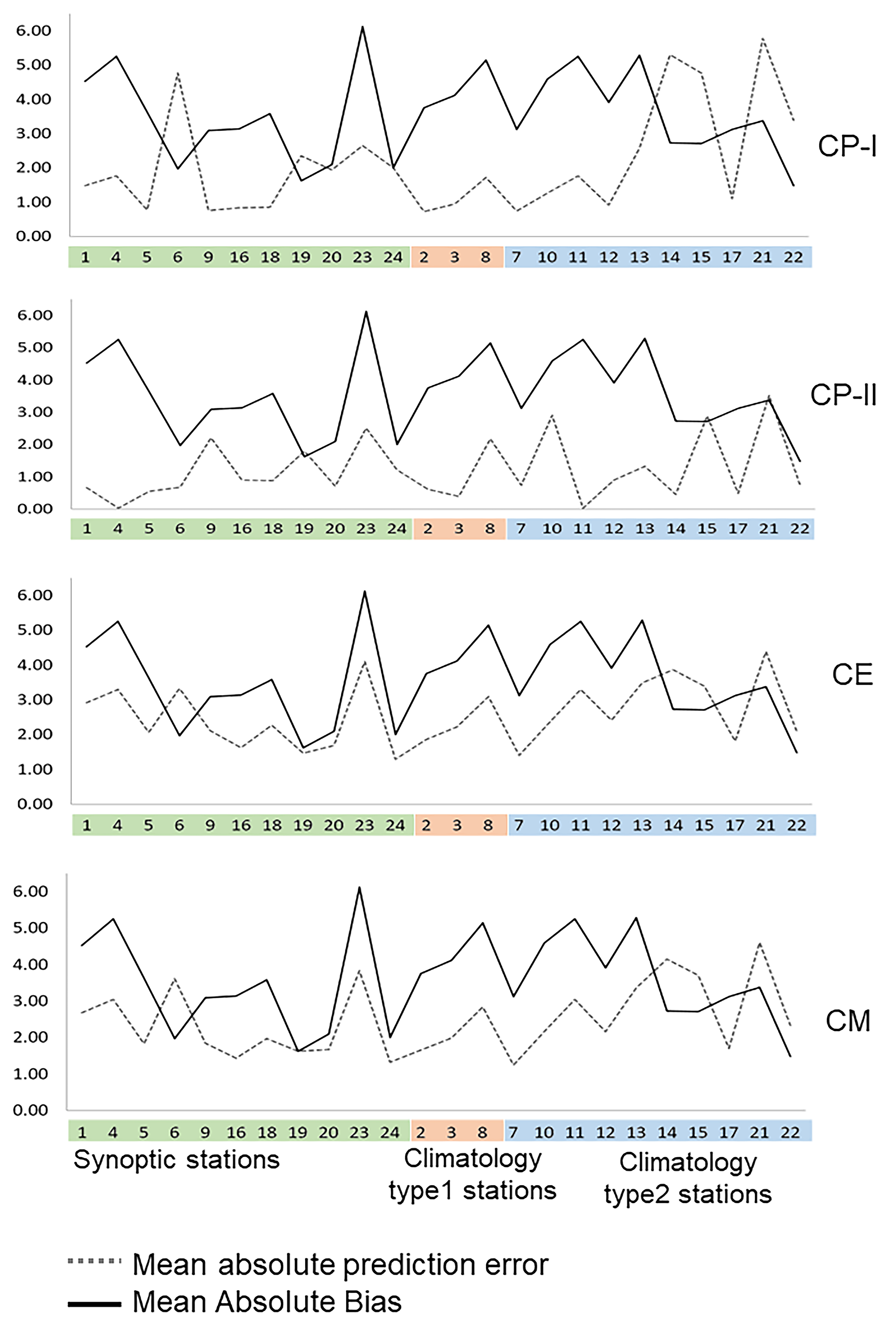

Supplement: S10 Fig — The vertical axis is error/bias in °C. The synoptic stations are supposed to provide more precise measurements. (TIF) [file pone.0216059.s015.tif]

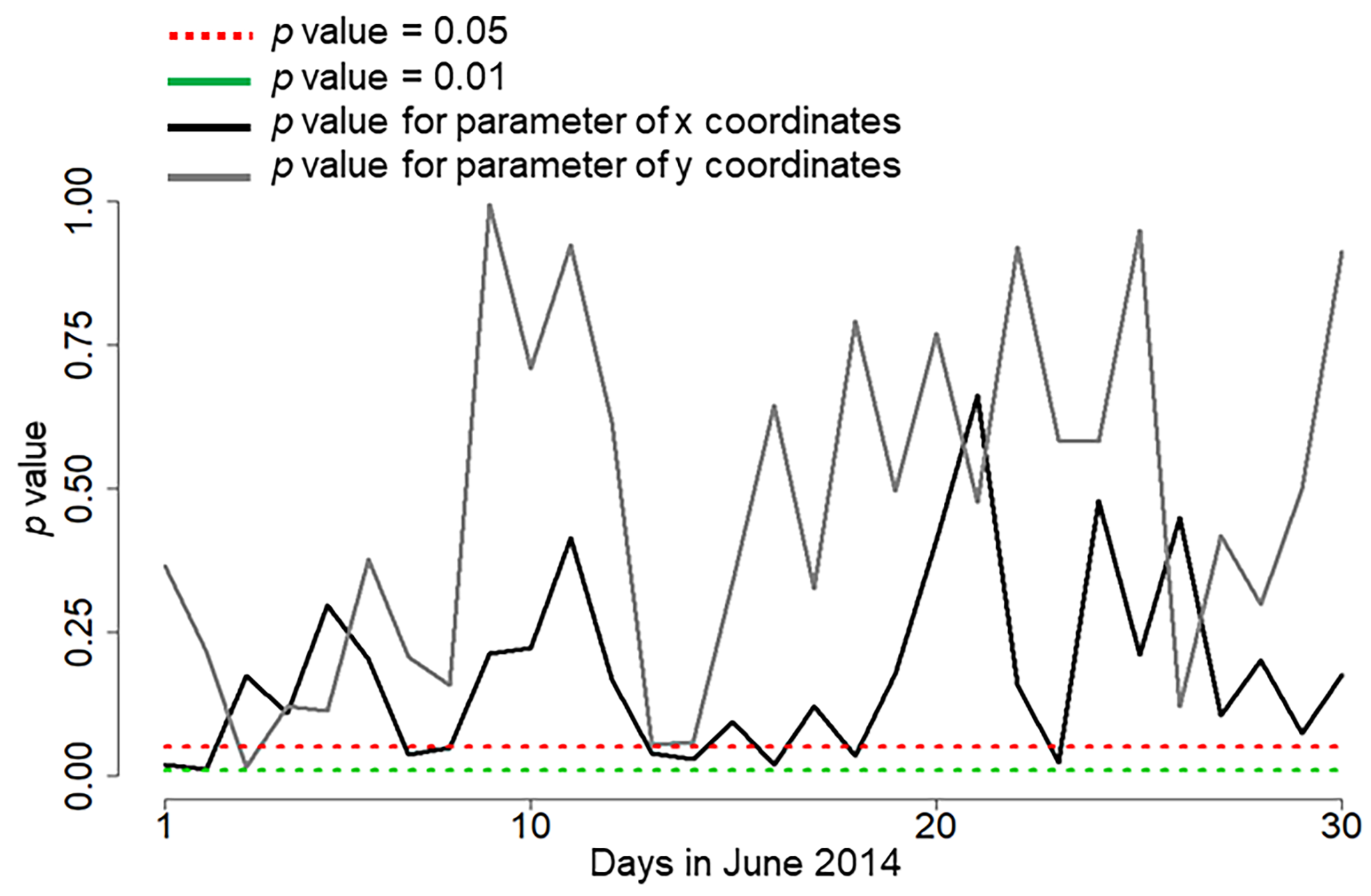

Supplement: S11 Fig — Based upon its results, spatial stationarity is assumed in estimating the marginal distribution. (TIF) [file pone.0216059.s016.tif]

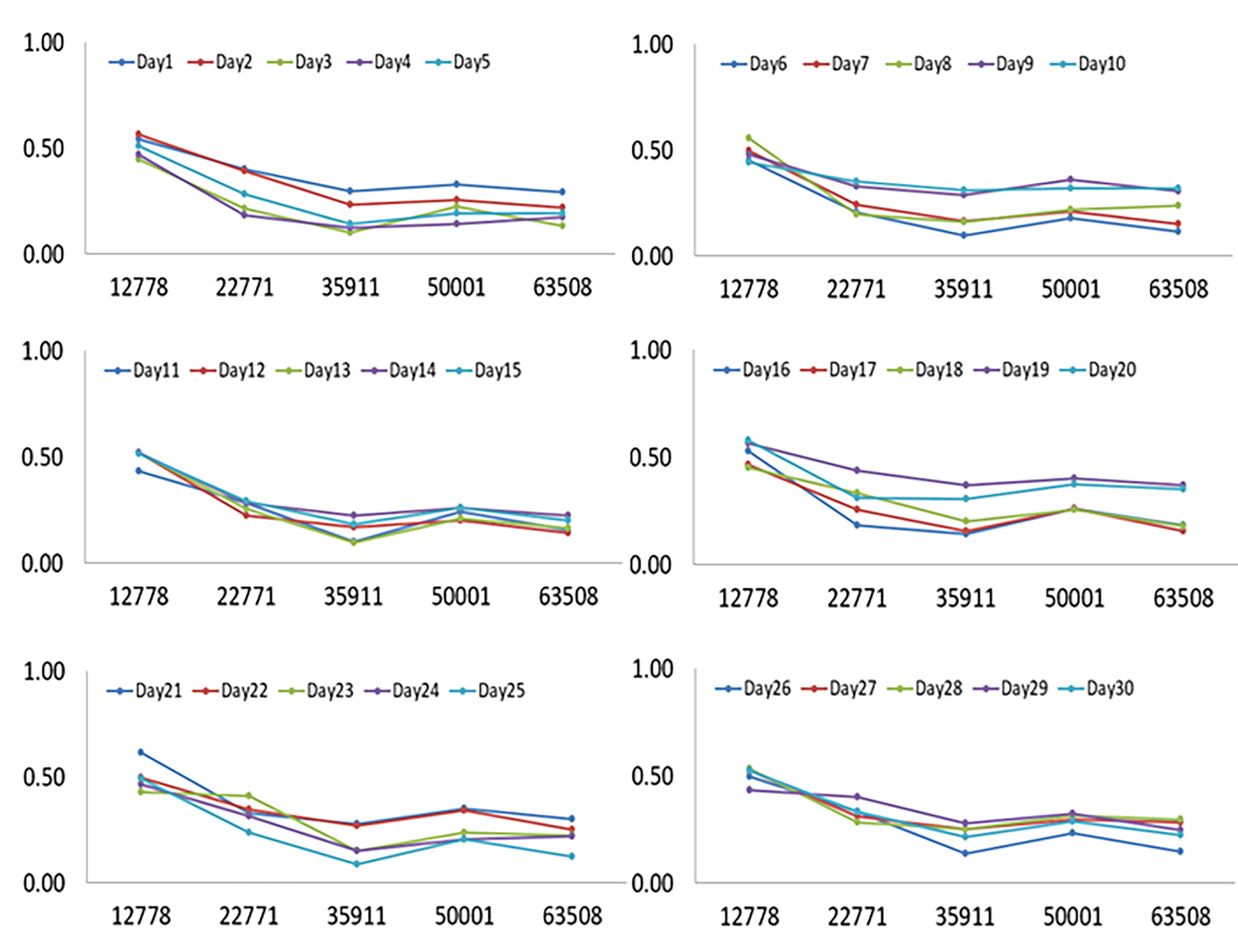

Supplement: S12 Fig — The vertical axis is Kendall’s τ correlations obtained using the measurements on each day in June between 2004 to 2014. The horizontal axis is spatial lags in meter. (TIF) [file pone.0216059.s017.tif]
